# Compostable Multilayer Films with Tailored Gas Barrier and Biodegradation

**DOI:** 10.1021/acsomega.5c09487

**Published:** 2026-03-06

**Authors:** Nasreddine Benbettaieb, Anibal Bher, Pooja C. Mayekar, Wanwarang Limsukon, Rafael Auras

**Affiliations:** 1 Food Processing and Microbiology, University Burgundy Europe, Institut AgroDijon, INRAE, UMR PAM, 1 Esplanade Erasme, Dijon 21000, France; 2 Bioengineering Dept., University Burgundy Europe, IUT-Dijon, 7 Blvd Docteur Petitjean, BP17867, Dijon Cedex 20178, France; 3 School of Packaging, 3078 Michigan State University, East Lansing, Michigan 48824, United States; 4 Department of Food Science and Technology, Faculty of Science and Technology, Rajamangala University of Technology Tawan-ok, Sriracha, Chonburi 20110, Thailand

## Abstract

This study investigates the barrier and biodegradation performance of reactive blends (reactive melt mixing inside the extruder) of poly­(lactic acid) [PLA] and thermoplastic cassava starch (TPCS). Two PLA-*g*-TPCS, plasticized with either glycerol (Gly) or poly­(ethylene glycol) (PEG), were prepared by twin-screw extrusion and processed into multilayer films through cast coextrusion. The films were characterized to assess structural, mechanical, thermal, optical, barrier, and surface properties, with emphasis on the effects of plasticizers. The biodegradation of these multilayer films was evaluated over 90 days using a direct-measurement respirometer system, tracking the evolution of CO_2_ in compost under thermophilic conditions. Fourier transform infrared spectroscopy (FTIR) analysis confirmed the successful grafting between PLA and TPCS. Reactive blending of PLA with TPCS significantly reduced tensile strength (TS) and Young’s modulus (YM) by over 50%, while elongation at break (EAB) increased by 70–80%. Incorporating the PLA-*g*-TPCS layer into a multilayer design tripled tensile strength and modulus compared to monolayers. TPCS enhanced chain mobility, lowering glass transition and melting temperatures. Gly-plasticized TPCS reduced oxygen permeability by 50%, whereas both plasticizers increased water vapor transmission and surface hydrophilicity. The hydrophilic TPCS accelerated abiotic degradation (hydrolysis of ester bonds due to humidity and temperature before biodegradation) of PLA under thermophilic conditions. Conversely, the PLA outer layers slowed the overall biodegradation rate in multilayer films, influenced significantly by the type of plasticizer used. Overall, PLA-*g*-TPCS multilayer films combine composability (in industrial facilities and possibly also in home composting environments) with functional performance, offering promise for sustainable packaging.

## Graphical abstract

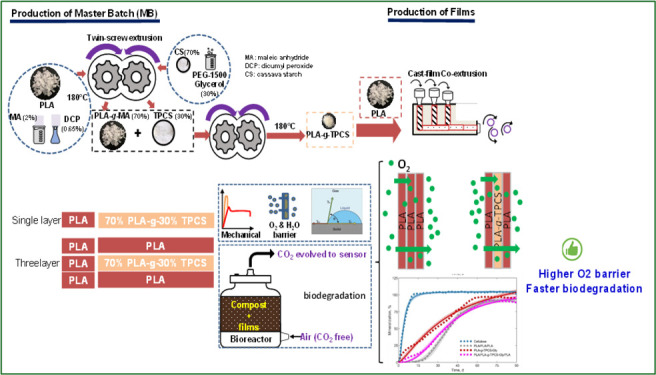

## 1 Introduction

Single-use plastics, predominantly made from fossil-based resources, are widely used in packaging due to their lightweight nature, cost-effectiveness, good processability, and low energy requirements during production and transportation.^1^ Furthermore, most of the flexible single-use plastic waste is derived from polyolefins, which can be recycled to some extent if clean and adequately collected. However, recycling plastics becomes difficult when they are contaminated with food or other residues. Replacing nonbiodegradable polymers with biodegradable ones that can be diverted with organic waste offers a promising solution to reduce single-use plastics destined for incineration or landfills. This shift could also support industrial composting by increasing organic waste diversion.^2^

Building on the need for alternatives to nonbiodegradable plastics, poly­(lactic acid) (PLA) has emerged as a sustainable option for food packaging. Derived from biobased sources, PLA offers desirable attributes such as decent water barrier properties, mechanical strength, transparency, and ease of processing.^3^ Approved by the U.S. Food and Drug Administration for food-contact applications,^4^ PLA stands out as a commercially viable biodegradable plastic. However, its limitations, including brittleness, poor oxygen barrier performance, low thermal stability, and limited degradation in environments other than thermophilic conditions, restrict its broader adoption. In this sense, PLA undergoes a phase of stubborn abiotic degradation, limiting its use in poorly managed industrial composting facilities. Some of these facilities perform rapid compost turning (approximately ≤2 months) and do not complete the whole mesophilic, thermophilic, and maturation phases required for PLA to biodegrade fully, resulting in residual material at the end of the process.^5^ This is a significant challenge given the limited availability of composting facilities that manage the full biodegradation of PLA, particularly in large urban areas. This infrastructure gap in the United States and other regions worldwide complicates the disposal of PLA and may undermine its environmental benefits; moreover, new regulations requiring single-use plastics to be home-compostable structures further constrain its application.^6,7^

To improve its performance for specific applications, PLA can be reactively blended with other biopolymers to enhance its gas barrier, conductivity, thermal, and overall physicochemical properties.^8^ These blended films can be recovered through composting. Polysaccharides and proteins are good candidates for developing sustainable blends and multilayer structures with PLA. Although proteins and polysaccharides tend to form hydrophilic structures with lower water vapor barrier performance and higher water sensitivity than PLA, they offer higher gas barrier properties in relatively dry environments.^9^ This complementarity suggests that multilayer structures, such as PLA-protein-PLA or PLA-polysaccharide-PLA, would not only protect the inner layer from water exposure but also enhance the barrier performance of PLA. Recent advances have demonstrated these benefits by creating blends or multilayer PLA structures with biopolymers such as cellulose,^10^ whey proteins,^11^ and gelatin.^12,13^ While these results are encouraging, with oxygen barrier efficiency increasing by more than 20 times, further efforts are needed to improve the compatibility and miscibility between the blends and to develop new strategies for commercially producing multilayer structures.

In some instances, PLA is still more expensive than conventional plastics, and its degradation rate remains slow compared to the rate of waste accumulation. One possible way to lower its price and improve its biodegradation rate is to introduce a low-cost filler, such as starch, into the PLA matrix.^14^

Thermoplastic starch (TPS) is a polysaccharide that can address some of PLA’s limitations in food packaging due to its oxygen barrier properties, biodegradability, abundance, and low cost.^15,16^ Blends of TPS or thermoplastic cassava starch (TPCS) and PLA can offer complementary properties, but their immiscibility, caused by poor interfacial adhesion, requires compatibilizers, such as maleic anhydride or peroxide initiators, to improve its overall performance.^15,17^

A bilayer assembly between PLA and starch enhanced the biodegradation of PLA, reducing the biodegradation lag period of PLA monolayer films.^18^ Heidemann et al.^19^ discussed various processing techniques such as coextrusion and lamination and highlighted the improvements in mechanical and barrier properties achieved through multilayer structures. Multilayer films with a combination of PLA with starch showed significantly higher water vapor barrier performance than a starch film, while the mechanical properties were similar to the PLA films.^19^ Moreover, multilayer systems have proven to be effective in enhancing gas barrier properties and accelerating degradation. Ordoñez et al.^20^ designed multilayers PLA/starch/PLA materials with adequate barrier capacity to oxygen and water vapor, useful to extend the food shelf life. To this end, PLA and starch were laminated as three-layer assemblies with two PLA outer layers to protect the internal starch layer from moisture. The authors showed that PLA provides the laminates with mechanical resistance and water vapor barrier capacity, while the internal starch layer was protected from moisture sensitivity by the external PLA sheets, providing the laminates with oxygen barrier capacity. From other study in the same multilayer structure (PLA and TPS), hydrophilic TPS layers provide oxygen barriers, while being protected by outer hydrophobic PLA layers, and help trigger biodegradation when in a compost environment.^5,17^ Ordoñez et al.^20^ displayed that the partial migration of glycerol and water molecules from the starch layer to the PLA matrix could modify the PLA’s tensile behavior in the multilayer. The high degree of sensitivity of PLA to hydrolysis in the presence of migrated water should largely enhance the biodegradation rate of the multilayer systems.

This design balances improved shelf life, enhanced degradation rates, and functional performance for food packaging applications. This work focuses on designing three-layer films with PLA as outer layers and a reactive PLA-*g*-TPCS as the inner layer, produced through a cast-coextrusion method emulating industrial processing. The intention was to answer a question that remains poorly understood: how do plasticizers influence the biodegradation of PLA in a multilayer structure? For this, we investigated the role of TPCS as an inner layer in the barrier performance and its effect on accelerating the biodegradation of multilayer PLA-based structures under thermophilic conditions in a compost environment. We monitored CO_2_ evolution throughout the test duration to replicate real-life scenarios encountered under industrial composting conditions.

## 2 Materials and Methods

### 2.1 Materials

Poly­(96% l-lactic acid) (PLA 2003D) resin was acquired from NatureWorks LLC (Ingeo, Minnetonka, MN, USA). Cassava starch (CS) was provided by Erawan Marketing Co., Ltd. (Bangkok, Thailand). Glycerol (>99.5% purity), polyethylene glycol (PEG-1500, melting point ≃ 45–50 °C), maleic anhydride (MA, >99% purity, *M*_w_ = 98.06 g/mol), and dicumyl peroxide (DCP, 98% purity, *M*_w_ = 270.37 g/mol, mp ≃ 39–41 °C, δ = 1.56 g/mL) were all purchased from Sigma-Aldrich (Milwaukee, WI, USA).

### 2.2 Production of Master Batches and Multilayer Cast Films

Before processing, the PLA resin was preconditioned at 50 °C for 12 h. The TPCS was obtained by mixing CS with glycerol or PEG (70/30% (w/w)) and extruding it into pellets. After processing, TPCS pellets were dried at 50 °C for 3 h (this temperature and time were selected to effectively remove residual surface and absorbed moisture from the TPCS pellets while avoiding starch degradation or retrogradation at higher temperatures). CS plasticized with glycerol or PEG (to produce TPCS) was extruded (temperature profile in Table 1) in a Century ZSK-30 twin-screw extruder (Century, Traverse City, MI, USA), pelletized in a BT 25 pelletizer (Scheer Bay Co., Bay City, MI, USA), and held in an oven at 50 °C for 3 h to remove residual water.

**Table 1:** Conditions of Processing of Master Batches in the Twin-Screw Extruder^a^

films	temperature profile from the feeder to the die (°C)	screw speed (RPM)
TPCS-Gly	130/140/150/150/150/150/150/150/150/150	120
TPCS-PEG	130/140/150/150/150/150/150/150/150/150	120
PLA-*g*-MA	160/170/180/180/180/180/180/180/170/160	120
PLA-*g*-TPCS-Gly	140/150/160/160/160/170/170/170/170/160	120
PLA-*g*-TPCS-PEG	140/150/160/160/160/170/170/170/170/160	120

a RPM: revolutions per minutes; PLA: poly­(lactic acid), TPCS: thermoplastic cassava starch, MA: maleic anhydride. Gly: glycerol, PEG: poly­(ethylene glycol).

PLA-*g*-TPCS was produced in two steps. During the first step, the PLA resin was mixed with 2% MA (w/w) and 0.65% DCP (w/w), based on PLA weight, and then extruded and pelletized (to produce the PLA-*g*-MA) according to the parameters presented in Table 1. The PLA-*g*-MA pellet was then stored in the oven at 50 °C for 3 h. In the second step, 70 wt % PLA-*g*-MA pellet was mixed with 30 wt % TPCS pellet and extruded and pelletized under the same method as PLA-*g*-MA.

This ratio was chosen as the best practical composition to achieve good dispersion of TPCS in the PLA matrix and stable melt processing behavior as previously demonstrated by Detyothin et al.^21^ and Detyothin et al.^22^

Master batches were used to produce cast films in a single extruder, whereas the three-layer films were coextruded using two single extruders (Randcastle Extrusion Systems, Inc., Cedar Grove, NJ, USA). Before the cast film process, all materials were conditioned overnight in an oven at 50 °C. The processing conditions of single and three-layers films are presented in Table 2. To minimize physical aging, the produced films were stored in a freezer at −4 °C until use for further characterization.

**Table 2:** Processing Cast Film Extrusion Conditions for Single- and Three-Layer Films^a^

films	extruder	temperature profile from feeder to the die (°C)	screw/roller speed (RPM)	chiller (°C)
PLA		177/177/182/182/182/182/182	15/20	30
PLA-*g*-TPCS-Gly		132/138/143/149/149/149/149	20/25	21
PLA-*g*-TPCS-PEG		132/138/143/149/149/149/149	15/20	18
PLA/PLA/PLA	extruder 1	182/182/182/182/182/116/116	15/20	24
	extruder 2	182/182/182/182/***/171/171	15/20	
PLA/PLA-*g*-TPCS-Gly/PLA	extruder 1	160/160/160/160/160/149/149	30/40	24
	extruder 2	149/149/149/149/***/149/149	25/40	
PLA/PLA-*g*-TPCS-PEG/PLA	extruder 1	182/182/182/182/182/149/149	30/40	24
	extruder 2	149/149/149/149/***/149/160	20/40	

a Winding roller speed: 12 rpm, extruder 1 used to produce both PLA-outer layers, and extruder 2 used to produce the reactive PLA-*g*-TPCS blend inner layer.

### 2.3 Film Characterization

#### 2.3.1 Film Thickness

The thickness of single-layer films was measured using a TMI digital micrometer (Testing Machines Inc., Ronkonkoma, NY, USA). The mean value was calculated using five measurements obtained from random locations of the film. The thickness of multilayer films was measured using a Keyence VHX-6000-950F digital microscope at the Center for Advanced Microscopy (Michigan State University, East Lansing), with a magnification range up to 5000×, and analyzed with VHX-6000-950F Measurement Data Tabulation Tool software using at least three positions in each image.

#### 2.3.2 UV–Visible and Fourier Transform Infrared (FTIR) Spectroscopy

The transmittance of the films (%T) was evaluated with a Shimadzu UV-1800 UV–visible scanning spectrophotometer in a wavelength range of 200–800 nm using one cycle and a medium scan speed (480 nm min^–1^). The FTIR spectra of single- and three-layer films were acquired using an IR Affinity-1S-FTIR instrument (Shimadzu, Columbia, MD) equipped with an attenuated total reflectance (ATR) attachment (PIKE Technologies, Madison, WI). The absorbance spectrum was measured at room temperature in the range of 500–4000 cm^–1^ using 64 accumulations (scans) and a resolution of 4 cm^–1^. The measured spectra were analyzed with Spectrum Suite software, and the spectra were expressed as absorbance. Only the shifts of peaks were considered for specific chemical groups interacting within the single-layer and multilayer films. All film samples were initially conditioned at 23 °C and 0% relative humidity (RH) for at least 7 days before analysis to prevent disturbance by −OH from water. Duplicate measurements for each sample were carried out.

#### 2.3.3 Mechanical Properties

Tensile strength (TS), elongation at break (EAB), and Young’s modulus (YM) were evaluated using an Instron Universal Machine 5565 (Instron, Norwood, MA, USA) according to ASTM D882–18.^23^ Film samples were cut into specimens with a width of 2.54 cm and a length of 20 cm considering the machine direction. Before testing, all the samples were equilibrated for 2 weeks in an environmental chamber (Environmental Growth Chambers, Chagrin Falls, OH, USA) at 23 °C and 50% RH. All films were tested with an initial grip and rate grip separation of 100 mm and 10 mm min^–1^, respectively. Twelve samples were evaluated for each formulation, and then, the average of each parameter was calculated.

#### 2.3.4 Differential Scanning Calorimetry (DSC)

A Q100 differential scanning calorimeter (TA Instruments, New Castle, DE, USA) was used to determine the glass transition temperature (*T*_g_), crystallization temperature (*T*_c_), melting temperature (*T*_m_), and initial degree of crystallinity (*X*_c_). Around 5 to 10 mg of each film was sealed in aluminum pans, cooled to 5 °C, remained isothermal for 3 min, then heated to 210 °C (first heating cycle), and held for 3 min to eliminate the thermal history during film production. Afterward, the system continued to the second cooling and heating cycle, where the sample was cooled to 5 °C and then heated to 210 °C (second heating cycle). The temperature ramp rate for all the cycles was 10 °C/min. The samples were tested in triplicate. Nitrogen purge flow was maintained at a constant rate of 70 mL/min. The initial degree of PLA’s *X*_c_ was determined according to eq 1 below.

$$X_{c}=\left[\frac{\Delta H_{m}-\Delta H_{c}}{\Delta H_{m}^{0}\left(1-\alpha \right)}\right]\times 100$$ 1

where Δ*H*_m_ is the heat of fusion, Δ*H*_c_ is the cold crystallization enthalpy, Δ*H*_m_^0^ is the heat of fusion of 100% crystalline PLA (93.0 J/g), and α is the sum of the weight fractions of TPCS, MA, and DCP in the final blends.

#### 2.3.5 Thermal Gravimetric Analysis (TGA)

TGA was conducted on a Q-50 thermogravimetric analyzer (TA Instruments, New Castle, DE, USA). Each sample (between 5 and 10 mg) was heated from room temperature to 600 °C at a ramp rate of 10 °C/min under a high-purity nitrogen atmosphere (70 mL/min). The degradation temperature (*T*_onset_ and *T*_dmax_) and residue were determined using the TA Universal Analysis 2000, V4.5 software.

#### 2.3.6 Dynamic Mechanical Analysis (DMA)

The viscoelastic properties of materials were studied using a TA RSA-G2 Solids Analyzer DMA unit (TA Instruments, New Castle, DE, USA) equipped with a tension geometry and frequency value of 1 Hz. The films were cut into strips (10 × 50 mm) in the machine direction and stored at 50% RH and 23 °C for 48 h in an environmental chamber. Three specimens were evaluated for each formulation. The storage modulus (*G*′), loss modulus (*G*″), and tan­(δ) were assessed. The initial distance between the two grips (loading gap) was 15 mm. A dry condition test (tension clamps) was performed, and the samples were heated from 25 to 100 °C at a constant rate of 5 °C/min, max gap changes up 5–10 mm, max gap changes down 1 mm, 0.2% strain, and preload force 100 g with a sensitivity of 10 g. The *T*_g_ of each sample was determined from the temperature at which the tan­(δ) peak was located.

#### 2.3.7 Molecular Weight Determination

The average molecular weight number (*M*_n_), average molecular weight (*M*_w_), and polydispersity index (PI) of samples were measured by size-exclusion chromatography (SEC) (Waters Corporation, Milford, MA, USA) using tetrahydrofuran (THF) (Sigma-Aldrich, St. Louis, MO, USA) as a solvent. For this test, around 10 mg of film was dissolved in 5 mL of HPLC grade THF (Pharmco-Aaper, Shelbyville, KY, USA) at ambient temperature and held for 1 day. In case of incomplete dissolution of films, the solution was heated at 40 °C for 12 h, cooled, and then filtered twice through a 0.2 mm poly­(tetrafluoroethylene) (PTFE) filter. The filtrate (100 μL) was injected into a Waters VR gel permeation chromatograph (GPC), with a Waters VR 1515 isocratic pump and a Waters VR 717 autosampler, and a Waters VR 2414 refractive index detector interface, with THF as a mobile phase at a flow rate of 1 mL/min (Waters Corporation, Milford, MA, USA). A series of Waters VR Styragel columns (HR4, HR3, and HR2) (300 × 7.8 mm [I.D.]) were used with a controlled temperature of 35 °C. The *M*_w_ or *M*_n_, and PI values were determined using Waters VR Breeze GPC software. Parameters *K* (0.0174 mL/g) and α (0.736) values of dilute PLA solution in THF at 35 °C were converted relative to absolute PLA *M*_w_.^17^ In the present study, *M*_n_ and *M*_w_ were determined for the final single- and multilayer films, not for the intermediate PLA-*g*-MA pellets, as the production optimization of PLA-*g*-MA and its reactive blending was previously conducted by the authors.^24,17^ The experiments were conducted in triplicate.

#### 2.3.8 Barrier Properties

The water vapor and oxygen barrier performances of the film samples were measured using Permatran model 3/33 and Ox-tran model 2/21 testing instruments (Mocon, Minneapolis, MN, USA), respectively. Samples were mounted in masks of aluminum foil (McMaster-Carr, Aurora, Ohio, USA) with an exposed area of 3.14 cm^2^. Accelerated test conditions at 90% RH and 37.8 °C were used for the water vapor permeability test according to ASTM F1249.^25^ The water vapor transmission rate (WVTR: g·m^–2^·d^–1^) was continuously monitored until a steady state was reached, and then, the water vapor permeability (WVP: g·m^–1^·s^–1^·Pa^–1^) was calculated according to eq 2.

$$\mathrm{WVP}=\frac{\mathrm{WVTR}}{\Delta p}\times t$$ 2

where Δ*p* is the difference in permeant partial pressure across the films expressed in Pa, and equal to the difference of water activity on both sides of the film (0.9 in this case) multiplied by water vapor pressure (*p* = 6560.9 Pa) at the test condition (37.8 °C), and *t* is the film thickness (m).

For oxygen permeability, two conditions were used: 0% and 50% RH at 23 °C and 1 atm, according to ASTM F1927–20.^26^ The O_2_ transmission rate (OTR: cc·m^–2^·d^–1^) was continuously monitored until a steady state was reached, considering an O_2_ concentration of 100%. The oxygen permeability (O_2_P: cc·m^–1^·d^–1^·Pa^–1^) was then calculated according to eq 3.

$$O_{2}P=\frac{\mathrm{OTR}}{\Delta p}\times t$$ 3

where Δ*p* is the difference in permeant partial pressure of oxygen across the films expressed in Pa, and is equal to the difference between *P*_atm_ (1.013 Pa) and the other side pressure, which is zero since the oxygen that permeates is continuously sent to the sensor; and *t* is the film thickness (m). For both barrier properties, at least four samples were tested for each type of film.

#### 2.3.9 Surface Properties

Water contact angle (WCA) measurement was used as a proxy for surface evaluation of the films. For the three-layer films, the WCA was determined for both side-faces of the outer layer: Face 1 (F1) and Face 2 (F2) were used to detect surface asymmetry and to understand how TPCS migration affects wettability differently on each side.

The WCA was measured using the sessile drop method on a goniometer (ramé-hart Instrument Co., Succasunna, New Jersey, USA) equipped with image analysis software (DROP image Advanced, ramé-hart Instrument Co.). A drop of demineralized water (∼2 μL) was deposited on the film surface using a precision syringe. The method is based on image processing for contact angle measurement from a theoretical meridian drop profile, measuring the contact angle between the baseline of the drop and the tangent at the drop boundary. About 20 values of the left and right contact angles were recorded during this period. The mean values of the left and right contact angles were then determined. Five measurements per film were carried out at 23 ± 2 °C and RH of 40% ± 5%. All films were equilibrated at 50% RH and 23 °C for 7 days before analysis.

#### 2.3.10 Biodegradation

Approximately 8 g samples of each film were cut into ∼1 cm^2^ pieces and placed in a 1.9 L bioreactor containing a mixture of manure-straw compost screened through a 10 mm mesh and sourced from the Michigan State University Composting Facility (East Lansing, MI, USA) and vermiculite that was pre-equilibrated with deionized water. The compost-to-vermiculite ratio was maintained at 4:1 (based on dry weight), adjusting the overall moisture content to 50%. CO_2_ evolution was measured for different films by using triplicate bioreactors, and results are presented as mean values with standard errors. An additional bioreactor was used for sampling to monitor the *M*_n_ and *M*_w_ reductions. Three blank bioreactors (containing only compost) served to assess background CO_2_ evolution, and three bioreactors containing 8 g of cellulose powder were included as a positive control. The physicochemical properties of compost were analyzed by the University of Missouri Soil and Plant Testing Laboratory (Columbia, MO, USA), with the results summarized in Table S1 of the Supporting Information.

Biodegradability was assessed under aerobic composting conditions using a direct measurement respirometer (DMR) system, with key parameters, temperature (58 ± 2 °C), RH (50% ± 5%), and airflow rate (200 ± 5 cm^3^/min), strictly controlled throughout the experiment. The methodology and equipment used are comprehensively described in a previous study.^27^ The CO_2_ evolved from the blank bioreactor (containing only compost) was considered as the background signal. This value was subtracted from the CO_2_ evolution measured in each sample bioreactor to determine the net CO_2_ production. The percentage of biodegradation was then calculated as the proportion of carbon from the sample converted to CO_2_, following eq 4:

$$\%\;\mathrm{of}\;\mathrm{biodegradation}=\frac{{\left({\mathrm{CO}}_{2}\right)}_{t}-{\left({\mathrm{CO}}_{2}\right)}_{b}}{M_{t}\times C_{t}\times \frac{44}{12}}$$ 4

In this equation, the numerator corresponds to the difference between the average cumulative mass of CO_2_ evolving from the three sample bioreactors (CO_2_)_t_ and the average CO_2_ evolving from the three blank bioreactors (CO_2_)_b_. The denominator represents the theoretical maximum amount of CO_2_ that could be produced from the complete mineralization of the sample’s carbon content. Here, *M*_t_ is the total mass of the sample, *C*_t_ is the proportion of carbon in the sample as determined by CHN elemental analysis, and 44 and 12 are the molecular weight of CO_2_ and the atomic weight of carbon, respectively.^28^ The carbon content for each film formulation was quantified using a PerkinElmer 2400 Series II CHNS/O Elemental Analyzer (Shelton, CT, USA), with the results presented in Table S2 in the Supporting Information.

The experimental degradation data were modeled using the Hill equation (eq 5):

$$Deg=\frac{{Deg}_{\max}\times t^{n}}{k^{n}+t^{n}}$$ 5

where *Deg* at time *t* (day) is the percentage of mineralization, *Deg*_max_ is the percentage of mineralization at infinite time, *k* (day) is the time when *Deg* = 1/2 *Deg*_max_, and *n* represents the curve radius of the sigmoid degradation function.

#### 2.3.11 Statistical Analysis

Statistical analysis was performed using SPSS 13.0 software (SPSS Inc., Chicago, USA). Analysis of variance (one-way ANOVA test) was performed to determine the significant difference through multiple comparisons of means. The least significant difference (LSD) mean comparison test was used at the significance level of 95% (*p*-value <0.05) to compare all the parameters analyzed between films.

## 3 Results and Discussion

This work aimed to evaluate the influence of a PLA-*g*-TPCS inner layer on the gas barrier performance, biodegradation behavior, and structural/physicochemical properties of multilayer PLA films while also proposing a mechanism to accelerate PLA biodegradation. Neat PLA films, single-layer reactive PLA-*g*-TPCS films, and three-layer PLA/PLA-*g*-TPCS/PLA films plasticized with Gly or PEG were produced. The multilayer design was selected to combine PLA’s hydrophobicity, mechanical strength, and durability with the oxygen barrier capacity and biodegradability of TPCS, overcoming the limitations of single-layer systems. The PLA-*g*-TPCS inner layer was engineered to improve compatibility, strengthening cohesion, and gas barrier performance compared to alternative inner layers. The hydrophobic PLA outer layers protect the hydrophilic core, preventing premature plasticization while maintaining functionality. At the same time, the plasticized TPCS inner layer enhances water diffusion, accelerating biodegradation. The effects of plasticizer chemistry, polarity, content, and molecular weight on thermal, mechanical, and barrier properties as well as on migration have been reported in the literature.^29^

### 3.1 Film Structural and Functional Properties

The thickness of single-layer and multilayer films is summarized in Table S3 of the Supporting Information, as determined by digital microscopy analysis. Figure 1a shows the appearance of single- and multilayer films and their %*T* values at 600 nm, indicating their transparency. The lowest transmission value indicated that the PLA-*g*-TPCS-Gly film was hazier than the other structures. Figure S1 shows the %*T* of all the films versus wavelength. All films, except PLA-*g*-TPCS-Gly, displayed a higher %*T* in both UV and visible light ranges. The PLA-*g*-TPCS-Gly films had the lowest %*T* (less than 55%) in the UV region, indicating an excellent barrier against UV light while also being efficient in the visible range. For a single layer, the %*T* values of PLA, PLA-*g*-TPCS plasticized with Gly, and PLA-*g*-TPCS plasticized with PEG were 82%, 68%, and 67%, respectively, at 600 nm. Transmittance was reduced in all the films produced with TPCS, compared to neat PLA, as also reported for the opacity of starch films, primarily due to the amylose content and distribution of starch in the film matrix. Bher et al. reported that reactive blend films (PLA-*g*-TPCS) had much lower %*T* values than physical blend films (PLA-TPCS) due to better distribution and compatibilization of the TPCS granules in the PLA matrix.^17^ Compared to neat PLA in the present study, the reduction in %*T* for the three UV regions (UV-C, 200–280 nm; UV-B, 280–320 nm; UV-A, 320–380 nm) is most pronounced in the case of glycerol-plasticized TPCS compared to PEG. The presence of PLA on both sides of the three-layer films increased the %*T* of PLA/PLA-*g*-TPCS-Gly/PLA and PLA/PLA-*g*-TPCS-PEG/PLA by 19% and 18%, respectively, at 600 nm. This result suggests that multilayer films based on PLA and PLA-*g*-TPCS are suitable for the manufacturing of transparent packaging films.

**Figure 1:**
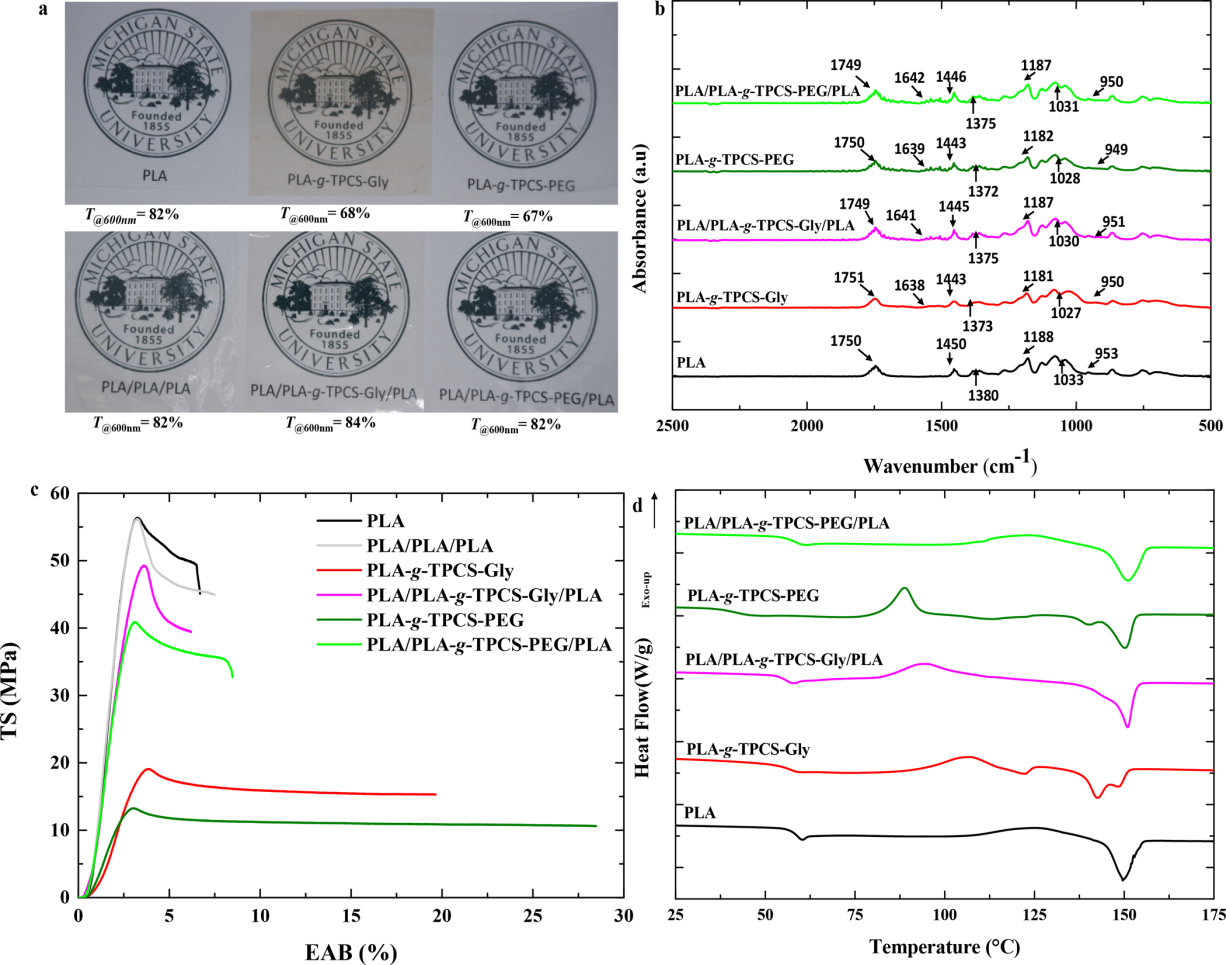
Characterization of PLA, PLA/PLA/PLA, PLA-*g*-TPCS-Gly, PLA/PLA-*g*-TPCS-Gly/PLA, PLA-*g*-TPCS-PEG, and PLA/PLA-*g*-TPCS-PEG/PLA films: (a) Visual appearance and transmission rate at 600 nm. Photograph courtesy of Dwi Yudison. Copyright 2026; (b) FTIR spectra in absorbance mode as a function of wavenumber (cm^–1^); (c) tensile properties (TS vs EAB) and (d) DSC thermograms of the second heating cycle of the films.

Figure 1b shows the FTIR-ATR spectra in the range of 500 to 2500 cm^–1^. The full FTIR spectrum (500 to 4000 cm^–1^) is presented in the SI­(Figure S2). For the PLA film, the bands at 2993 and 2947 cm^–1^ are assigned to the asymmetric and symmetric stretching vibrations of the CH group. At 1750 cm^–1^, the typical asymmetric stretching of the carbonyl group (C=O) in the PLA film is attributed to lactide (ester group).^30^ The peaks at approximately 1450 and 1380 cm^–1^ are assigned to the asymmetric and symmetric −CH_3_ (lactide −CH_3_ group) deformation vibrations, respectively.^31^ A typical absorption for the asymmetric stretching of the C–O bond in the ester group (−COOR) appears at 1188 cm^–1^,^32^ and the bands at 1127 cm^–1^, 1084 cm^–1^, and 1033 cm^–1^ correspond to the stretching vibrations of the asymmetric C–O–C band, and the bands at 953 and 924 cm^–1^ are characteristic of vibrations of a helical structure with an oscillation of the CH_3_ group.^33^ The bands at 867 and 754 cm^–1^ are related to the stretching vibration of the C–C bond and correspond to the amorphous phases and crystalline PLA, respectively, thus indicating a semicrystalline PLA.^34^ New bands that are related to TPCS appeared in the PLA-*g*-TPCS films. The absorption bands at 3285 cm^–1^ (not shown) are attributed to the stretching vibrations of OH groups (of amylose, amylopectin, glycerol, and absorbed water).^33^ Another new broad band with small intensity was observed at 1648 cm^–1^, which is attributed to the vibrational bending mode of water molecules that absorb strongly, along with the hydroxyl groups, in the amorphous regions of the starch. Other relevant bands are located at 2920 cm^–1^ due to the CH groups at carbon 6 of the starch glucose units.^35,36^ Finally, the band at 1050 cm^–1^ corresponds to the C–O–H bending; this band is important due to changes in the starch structure, such as retrogradation.^33^ This band also corresponded to the glycerol (−OH group) or PEG added as a plasticizer. With the addition of TPCS, the stretching of PLA’s −CH could overlap with starch’s strong −CH stretching at 2993 cm^–1^, as previously observed by Trinh et al.^37^ This peak has a minimal intensity for the neat PLA film and the three-layer films, but the peak intensity increases in the case of single-layer films (PLA-*g*-TPCS) and shifts to a lower wavenumber. The same behavior was observed for the 2947 cm^–1^ peak (assigned to symmetric stretching vibrations of the CH group). The sharp absorption peak at 1750 cm^–1^ appears to shift to a lower wavenumber, which is observed in all films and is attributed to the grafting of PLA oligomers onto cassava starch. Similar results were found by Nazrin et al.^31^ at 1745 cm^–1^ and attributed to the possible interaction between these two immiscible polymers. According to Wang et al.,^38^ the carbonyl groups also play a role in the interaction between PLA and TPCS, increasing the dispersion of the two phases. With the addition of TPCS, the peaks at 1450 and 1380 cm^–1^ (lactide −CH_3_ group) can be seen to have shifted to a lower wavenumber. The same tendency was observed for the asymmetric stretching of the C–O bond in the ester group (−COOR), which appears at 1188 cm^–1^, and the bands at 1127 cm^–1^, 1084 cm^–1^, and 1033 cm^–1^ correspond to the C–O stretching vibration, as also shown by Nazrin et al.^31^ Figure 1b also shows that all films have predominant functional groups of hydroxyls. In contrast, the neat PLA film has the largest number of carbonyl groups. Peaks at 1638 cm^–1^, assigned to absorbed water molecules on the starch structure, declined in intensity with the grafting of PLA into TPCS, which could be associated with increased hydrophobicity. The FTIR analysis of PLA-*g*-MA confirmed the successful grafting of maleic anhydride (MA) onto the PLA backbone, as evidenced by characteristic anhydride-related absorption bands while also suggesting the presence of residual oligomeric or unreacted MA species. Previous titration measurements indicated a maximum grafted MA content of 0.52 wt %, achieved at the expense of a significant reduction (≈50%) in the number-average molecular weight (*M*_n_) and a concomitant increase in polydispersity index to approximately 2.0, highlighting the occurrence of chain scission during reactive extrusion. In general, increasing the peroxide initiator (L101 or DCP) content enhanced grafting but also promoted molecular weight degradation.^39^ Both L101 (or DCP) concentration and MA grafting level strongly influenced the stability of *M*_n_ during storage, suggesting that residual reactive species or oligomeric MA may continue to affect chain integrity postprocessing. Under nominal conditions, we have demonstrated that an MA content of 4.5 wt %, L101 between 0.45 and 0.65 wt %, and a screw speed of 20 rpm provide an optimal compromise between grafting efficiency and molecular weight retention.^39^ It should be noted that beyond the effects of grafted and free MA, glycerol and PEG introduced as plasticizers within the TPCS phase may also contribute to increased water uptake, enhanced hydrolysis, and modified gas transport properties of the resulting films. Because control samples consisting of PLA plasticized with glycerol or PEG in the absence of TPCS were not included, the individual contributions of plasticizers, thermoplastic starch, and MA-related species cannot be fully decoupled. Nevertheless, the combined effect of TPCS and plasticizers reflects a realistic and industrially relevant formulation strategy for biodegradable packaging materials. This limitation is acknowledged and will be addressed in future studies through dedicated control formulations that quantitatively isolate the role of each component. Furthermore, future work will focus on the further quantitative determination of MA partitioning and its specific interactions within the PLA/TPCS system.

Figure 1c illustrates the TS as a function of the EAB for single- and three-layer films produced via cast film extrusion. PLA films are rigid and brittle, with three-layer PLA films exhibiting similar tensile properties to single-layer PLA films. Blending PLA with Gly or PEG-plasticized TPCS reduced TS and YM by over 50% while increasing EAB by approximately 70–80% (Table S4). This result indicates that PLA-*g*-TPCS films are more flexible but less strong and stiff than neat PLA, due to the ductility of plasticized TPCS.^40^ The reduced rigidity and increased deformability in reactive films result from the elastic and hygroscopic nature of TPCS, which is further enhanced by the plasticization of Gly or PEG. Reactive blending improved compatibility between PLA and TPCS, increasing EAB by more than 71% and reducing TS by more than 68% compared to neat PLA (Table S4), consistent with previous findings.^17^ Gly-plasticized TPCS produced films with greater rigidity and strength than PEG-plasticized TPCS due to the finer morphology and stronger bonds formed by glycerol with starch molecular chains, which restrict chain mobility. In contrast, the tendency of PEG to better lubricate the PLA matrix during extrusion weakened the interfacial adhesion in the blend, reducing film strength and stiffness, as shown in previous work.^41^ For multilayer films, the PLA outer layers on both sides of the PLA-*g*-TPCS inner layer significantly enhanced tensile strength (TS) and Young’s modulus (YM) (by more than 3-fold), while markedly reducing elongation at break (EAB) (also by more than 3-fold), compared to PLA-*g*-TPCS single-layer films (Figure 2 shows the thicknesses of the two outer PLA and the thickness of the inner PLA-*g*-TPCS layer, as detailed in Table S3 (Supporting Information)). This behavior highlights the superior mechanical resistance of the multilayer architecture, in agreement with previous reports on similar PLA-based multilayer systems.^42^ Therefore, optimizing EAB could be achieved by tailoring the thickness of the middle layer, allowing a compromise between the stiffness and flexibility of the multilayer films.

**Figure 2:**
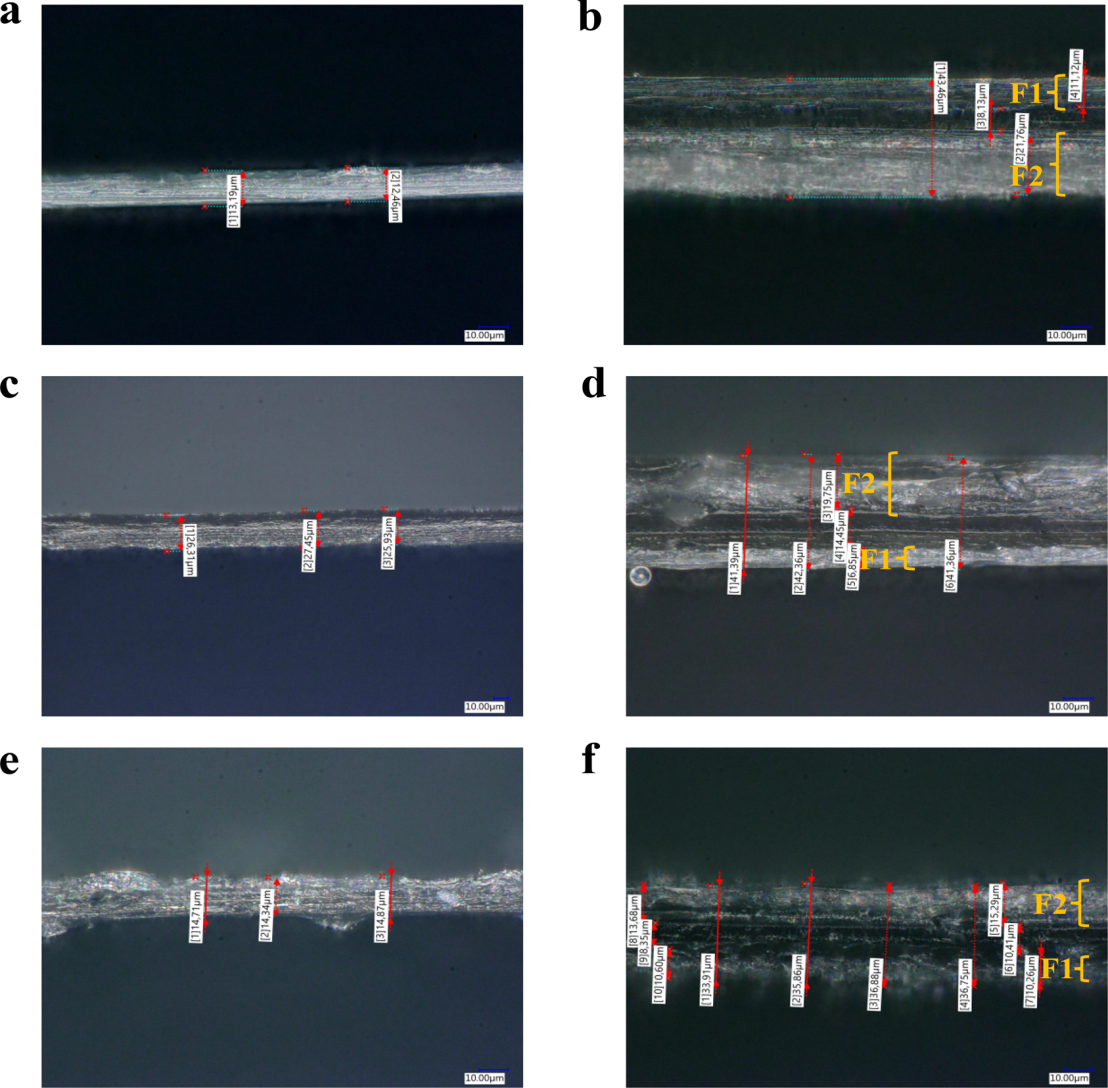
Visual appearance of the cross-section of single and multilayer films: (a) PLA, (b) PLA/PLA/PLA, (c) PLA-*g*-TPCS-Gly, (d) PLA/PLA-*g*-TPCS-Gly/PLA, (e) PLA-*g*-TPCS-PEG, and (f) PLA/PLA-*g*-TPCS-PEG/PLA.

Previous studies^21^ have shown that although increased MA grafting promotes interfacial bonding with thermoplastic starch, excessive grafting is accompanied by significant molecular weight reduction due to chain scission, which negatively affects tensile properties. In particular, PLA-*g*-MA with *M*_n_ values around 45 kDa and moderate grafted MA levels (∼0.1 wt %) were reported to provide optimal elongation at break in PLA/TPCS reactive blends.^21^ In the present work, PLA-*g*-MA was selected within a similar molecular weight range to balance the interfacial reactivity and mechanical integrity of the multilayer films.

To provide context for the characteristics of the PLA-*g*-MA used in this work, we refer to a previous study conducted by the same research group,^24^ in which PLA-*g*-MA was synthesized under comparable experimental conditions. In that study, PLA-*g*-MA was prepared with different MA contents (0, 0.5, 1, 1.5, 2, 2.5, 3, and 3.5 wt %) and with 0.1 and 0.2 wt % DCP. The coauthors reported a decrease in *M*_n_ and *M*_w_ of PLA-*g*-MA samples up to 0.5 wt % MA, while *Đ* remained relatively stable across both DCP contents. This moderate decrease (up to ∼25%) was attributed to the formation of PLA radicals induced by DCP. At MA contents above 0.5 wt %, MA branching and possible cross-linking reactions were suggested, leading to a slight increase in *M*_n_ and *M*_w_. Based on these findings and given that PLA-*g*-MA was produced using 2 wt % MA and 0.65 wt % DCP, it was reasonable to assume that MA grafting did not significantly reduce PLA molecular weight at the employed MA concentration. The resulting PLA-*g*-MA pellets were dried at 50 °C for 3 h and subsequently blended with 30 wt % TPCS (70 wt % PLA-*g*-MA/30 wt % TPCS). The mixture was then extruded and pelletized, using a procedure similar to that used for PLA-*g*-MA synthesis, to obtain PLA-*g*-TPCS. The *M*_n_, *M*_w_, and *Đ* values of PLA-*g*-TPCS-Gly and PLA-*g*-TPCS-PEG are provided in Table S5 of the Supporting Information. A reduction in *M*_n_ and *M*_w_ was observed after processing, consistent with the effects of MA and DCP concentrations and with the additional thermal and mechanical processing steps reported in the cited literature.

In another related study by the coauthors,^17^ it was shown that introducing PLA, TPCS, MA, and initiator simultaneously (procedure A) limited the reductions in *M*_n_ and *M*_w_ to less than 15%, due to reduced thermal history. In contrast, producing PLA-*g*-MA first and subsequently blending it with PLA and TPCS (procedure B) resulted in more pronounced reductions in *M*n (∼30%) and *M*w (∼18%), along with an increase in *Đ*, owing to the additional processing step. In both procedures, the reductions in *M*_n_ and *M*_w_ were attributed to free-radical formation from initiators such as DCP or L101, as well as to PLA chain scission resulting from hydrolysis reactions catalyzed by water and glycerol in TPCS. These studies also demonstrated that using MA as a compatibilizer significantly improved interfacial adhesion between PLA and TPCS, with a more pronounced effect when DCP was used as the initiator rather than L101, due to MA’s high reactivity with PLA’s terminal carboxyl and hydroxyl groups. By processing the films under the conditions used in this work, we achieved a balance between grafting and minimal *M*_n_ reduction was achieved.

Figure 1d illustrates the second DSC heating cycle, including *T*_g_, *T*_c_, and *T*_m_. The second heating scan was intentionally analyzed to eliminate the influence of thermal history and to enable a more direct comparison of intrinsic crystallization behavior among the samples, so we only focus on the second heating DSC results (the first heating cycle process is provided in Section S4-Page S11 of the SI). The neat PLA film exhibited a *T*_g_ of 57.2 °C, slightly lower than the value (61 °C) reported by Bher et al.^17^ and the value found from the DMA analysis (61.8 °C; Figure S3). PLA-*g*-TPCS films plasticized with glycerol and PEG showed *T*_g_ reductions of 3 and 16 °C, respectively, attributed to the plasticizing effects, which enhance polymer chain mobility.^21^ This shift in *T*_g_, reported in similar studies, is linked to increased free volume resulting from plasticizer migration and decreased polymer ordering and molecular weight, as shown in Table S5, which indicates a significant reduction in PLA’s *M*_n_ for PLA-*g*-TPCS-Gly and PLA-*g*-TPCS-PEG. As seen in the DSC thermograms, multilayer films displayed a single *T*_g_ value, ranging between neat PLA and PLA-*g*-TPCS (Figure 1d). The *T*_c_ for neat PLA was 125 °C, whereas PLA-*g*-TPCS films had significantly lower *T*_c_ values, reflecting increased chain mobility and nucleation effects from PLA-*g*-MA and DCP. These results align with the findings of Bher et al.,^17^ who observed a marked decrease in *T*_c_ for reactive PLA-*g*-TPCS films. *X*_*c*_ was low for neat PLA, consistent with prior reports, but significantly higher for PLA-*g*-TPCS films, particularly in the three-layer structures (Table S6). A double melting peak was observed in PLA-*g*-TPCS films plasticized with glycerol or PEG, probably attributed to α and α’ crystal formation, as noted by Detyothin et al.^21,44^ These crystal forms result from disrupted crystallization, with α’ representing an imperfect phase of α.^43^ The *T*_m_ of PLA-*g*-TPCS films decreased by 3 to 10 °C compared to neat PLA and three-layer films, reflecting these crystallization changes.

The films’ viscoelastic properties (*G*′, *G*″, and tan δ) reveal key effects of the TPCS phase and plasticizers (Figure S3). Adding TPCS reduced *G*′ (30–60 °C) compared to neat PLA, indicating decreased stiffness and elasticity due to increased chain mobility and flexibility.^17,21^ Reactive blends exhibited lower *G*′, particularly for PEG-plasticized TPCS, where PEG migration into the PLA outer layer significantly decreased stiffness. In comparison, glycerol-plasticized TPCS maintained higher G′ due to a stronger interaction with cassava starch. Above *T*_g_ (55–60 °C), *G*′ values for all films converged, with multilayer films showing higher *G*′ due to the PLA outer layers. *G*″ was reduced in reactive blends, reflecting less energy dissipation and tougher behavior. Tan δ peaks shifted below PLA’s 61 °C *T*_g_ for PLA-*g*-TPCS films, reflecting enhanced chain mobility and the plasticizing effects of glycerol or PEG.^37^ These results align with the mechanical and DSC findings.

The neat PLA film degraded in a single step (Figure S4), with a maximum degradation peak (*T*_dmax_) at 367 °C and negligible residue, consistent with prior findings.^30^ PLA-*g*-TPCS films exhibited four degradation steps: free water evaporation (110–200 °C), bound water and glycerol evaporation (200–300 °C), starch decomposition (300–320 °C), and PLA decomposition (320–370 °C), aligning with *T*_dmax_ values of individual components.^40^ The *T*_onset_ significantly decreased for PLA-*g*-TPCS, with glycerol-plasticized TPCS showing the lowest values, attributed to plasticizer migration and moisture-induced depolymerization.^17^ It has been reported that the initial moisture content of the starch had a significant effect on the decomposition of PLA during the processing of PLA/starch blends; the PLA hydrolyzes and degrades in the presence of water under the high levels of heat used during processing.^45^ PEG-plasticized TPCS exhibited better thermal stability than glycerol, reflecting the higher stability of PEG. An additional small peak at 450 °C in PLA-*g*-TPCS films was likely due to grafted PLA oligomer degradation, as reported by Trinh et al.^37^ Multilayer films with PLA outer layers increased *T*_onset_ and *T*_dmax_, demonstrating enhanced thermal stability as the PLA layers may shield the matrix from the effects of diffused plasticizers.

Table 3 illustrates the oxygen (O_2_P) and water vapor permeability (WVP) of single-layer and three-layer films. Producing high-oxygen and water-barrier compostable films is still out of reach. However, Table 3 shows that some improvement in the oxygen barrier can be obtained by introducing PLA-*g*-TPCS as an inner layer. Neat PLA films had O_2_P values of 3.84 ± 0.4 and 5.15 ± 0.08 × 10^–7^ cc/m·d·Pa at 0% and 50% RH, respectively. These values agreed with the value of 8.7 × 10^–18^ kg·m/m^2^·s·Pa (5.26× 10^–7^ cc/m·d·Pa) reported in the literature for PLA films exposed to the same RH condition.^46^ Blending PLA with 30% glycerol-plasticized TPCS reduced PO_2_ by up to 5-fold compared to neat PLA films (Table 3). For multilayer films, the improved barrier properties of the PLA/PLA/PLA multilayer film compared to the single PLA structure may be attributed to the layered structure, which may increase tortuosity at the interface and hinder the diffusion path for gases and also for moisture. Additional work needs to be conducted to understand the impact of the interphase. Similar effects have been reported in multilayer polymer films, where repeating layers of the same polymer improve barrier performance by creating a more complex diffusion path.^47^ Furthermore, the PLA-*g*-TPCS inner layer with glycerol reduced PO_2_ by 50% compared to single-layer or three-layer PLA films, enhancing oxygen barrier properties (Table 3). Similar improvements in oxygen barrier properties were observed in PLA-gluten-PLA films, with up to a 20-fold O_2_P reduction,^48^ and PLA-fish gelatin-PLA films.^49^ Hydrocolloids, like TPCS, offer higher O_2_ barrier properties than polyesters, making the multilayer system more effective. The outer PLA layers provide hydrophobic protection, preventing plasticization of the inner PLA-*g*-TPCS layer, while the TPCS inner layer significantly enhances the oxygen barrier properties. Additionally, improvement to the WVP is still needed. As the current study did not include control samples containing PLA with plasticizers only, we cannot fully distinguish the individual contributions of TPCS and the plasticizers to the observed barrier properties results. However, some of the observed effects may arise partially or wholly from the plasticizers themselves.

**Table 3:** Oxygen Permeability (O_2_P) and Water Vapor Permeability (WVP) of Single- and Three-Layer Films Produced^a^

	O_2_P (×10^–7^ cc/m·d·Pa) at 23 °C	WVP (×10^–6^ g/m·d·Pa) at 37.8 °C	
formulation	0% RH	50% RH	90% RH
PLA	3.84 ± 0.40^a^	5.15 ± 0.08^a^	1.67 ± 0.06^a^
PLA-*g*-TPCS-Gly	0.69 ± 0.00^b^	4.82 ± 0.94^b^	2.06 ± 0.08^b^
PLA-*g*-TPCS-PEG	6.32 ± 0.01^c^	8.23 ± 0.62^c^	3.05 ± 0.23^c^
PLA/PLA/PLA	2.18 ± 0.08^d^	5.00 ± 0.48^a,^ ^b^	1.35 ± 0.43^a^
PLA/PLA-*g*-TPCS-Gly/PLA	2.20 ± 0.04^d^	2.50 ± 0.86^d^	2.79 ± 0.07^c^
PLA/PLA-*g*-TPCS-PEG/PLA	3.98 ± 0.14^a^	3.99 ± 0.78^b^	2.89 ± 0.08^c^

a Mean ± std deviation. Values in a column with the same superscript letter are not significantly different at *p*-level = 0.05.

In the literature, Shin et al. indicated that the addition of PEG to PLA can significantly modify oxygen permeability and gas transport behavior, depending on PEG molecular weight and concentration.^50^ From this reference, the oxygen transmission rate (OTR) of PLA/PEG blend films generally decreases with increasing PEG content, which has been attributed to an increase in crystallinity and higher packing density due to interactions of PEG’s hydrophilic hydroxyl groups with PLA, as they evidenced by XRD measurements.^50^ At the same time, low-molecular-weight PEG can increase free volume in the polymer matrix, promoting permeation while also introducing polarity effects that reduce sorption of nonpolar oxygen molecules. Thus, plasticizers can contribute to both enhanced barrier performance and altered biodegradation behavior.^50^

The WVP of PLA single-layer films was 1.67 ± 0.062 × 10^–6^ g/m·d·Pa. Blending PLA with glycerol or PEG plasticized TPCS increased the WVP by 44%, depending on the plasticizer used. Grafting 30% TPCS into the PLA matrix increased WVP due to the hydrophilic nature of cassava starch, which allows water to saturate the surface, penetrate the structure, and be absorbed by starch, leading to higher water permeation. Noivoil and Yoksan reported that TPCS significantly decreases the water barrier properties of PLA/TPCS blends due to its inherent hydrophilicity and sensitivity to moisture.^40^ Nazrin et al.^51^ observed that substituting 20% TPS with PLA increased the WVP of PLA-TPS blends by over 90%. The hydrophilic nature of proteins and hydrocolloids further reduces the water barrier properties in multilayer films.^37^ However, introducing PLA to PLA-*g*-TPS blends improves the moisture barrier properties of neat TPS films, which are highly sensitive to water.^52^ Additionally, plasticizers like glycerol or PEG increase chain mobility and water transfer with PEG having a greater effect on WVP than glycerol.

Including a compatibilizer during twin-screw extrusion improved the compatibility of the PLA and TPCS phases by reducing interfacial spaces and limiting water diffusion compared to nongrafted systems. However, the PLA outer layers in the three-layer PLA/PLA-*g*-TPCS/PLA films did not enhance the WVP of the inner PLA-*g*-TPCS layer, contrasting with the findings from PLA-fish gelatin-PLA and PLA-gluten-PLA films,^12,48^ where hydrophobic PLA layers reduced interlayer plasticization and WVP. However, these cases involved the addition of proteins and not carbohydrates.

Although practical food-packaging simulation experiments, such as food preservation tests, would provide direct validation of the films’ barrier performance under real conditions, the primary objective of this study was to investigate the intrinsic structural, thermal, mechanical, and barrier properties of the multilayer films under controlled laboratory conditions. The incorporation of real-food-packaging simulations is therefore beyond the scope of the present work and will be addressed in future studies. The characterization of the WCA of the three-layer films revealed differences in wettability on both sides (Face 1 [F1] and Face 2 [F2]), which are linked to the migration of the plasticized TPCS inner layer (Table S7). PLA films had a WCA of 73 ± 1°, consistent with prior studies.^53^ Blending PLA with 30% TPCS reduced WCA by 6 to 10° due to the hydrophilic nature of starch and plasticizer effects.^37^ For three-layer films, one side resembled PLA-*g*-TPCS’s WCA, while the other matched neat PLA, suggesting higher plasticizer migration (Gly or PEG) to the F1 side. Similar behaviors were reported for a PEG-plasticized PLA through a reactive extraction approach,^54^ for PLA-TPS blends,^33^ and PLA-fish gelatin-PLA multilayer films.^12^ Furthermore, from the visual appearance of the cross-section of the single and multilayer films (Figure 2), we can clearly see that F1 is less thick than F2, so the migration of Gly or PEG takes place from F1 rather than from F2 and plays a plasticization role in that surface, which can explain why the WCA of the F1 side is lower than that of the F2 side. Figure 2 shows also layer adhesion and uniformity and layer thickness measurements, as discussed before.

### 3.2 Biodegradation in Simulated Composting Conditions

Here, we present the effect of PLA-*g*-TPCS as the inner layer on the biodegradation of the multilayer structure in simulated composting under thermophilic conditions (58 ± 2 °C). CO_2_ evolution and biodegradation of different structures were evaluated alongside PLA and PLA/PLA/PLA to understand the effect of reactive blending TPCS and the plasticizer nature on PLA degradation. The cumulative CO_2_ evolution and % biodegradation results during 90 days of testing are presented in Figure 3.

**Figure 3:**
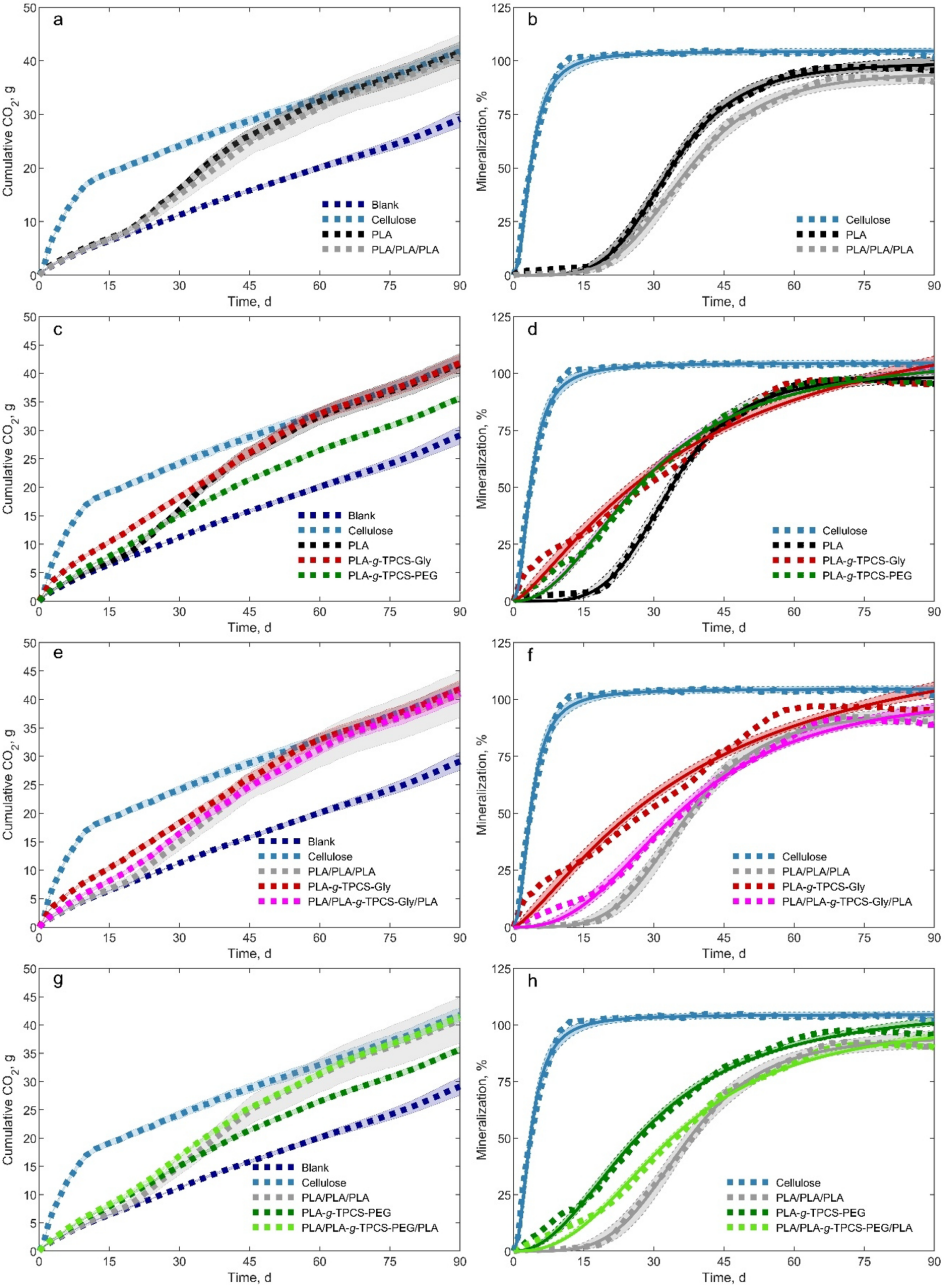
CO_2_ evolution and mineralization of single-layer and multilayer films. Dotted lines (···) represent average values across replicates. Shaded regions in (a), (c), (e), and (h) show the standard error of the mean (*n* = 3), while those regions in (b), (d), (f), and (g) show 95% confidence intervals obtained via bootstrapping of the residual of the fitted Hill equation. Mineralization values were estimated from eq 4.

Figure 3a,b shows the CO_2_ evolution and % biodegradation results, respectively, for blank (only compost), cellulose, PLA-single layer [SL] (PLA), and PLA-three layers [3L] (PLA/PLA/PLA). Table S8 summarizes the % biodegradation data for each sample on days 45, 60, and 90.

#### 3.2.1 Blank and Cellulose

The blank showed a maximum CO_2_ evolution of 30 g at day 90 (Figure 3a). The positive control cellulose achieved 42 g of CO_2_ evolution and showed a maximum biodegradation of 100%. Cellulose reached 100% biodegradation by day 12 without a lag phase, as it is a readily available food source for microorganisms and is easily biodegradable. Cellulose’s hydrophilic nature, combined with the activity of naturally occurring enzymes, facilitates its degradation, allowing it to pass through microbial cell walls and be readily assimilated via metabolic pathways.^55^ Cellulose is broken down by a complex group of enzymes that act simultaneously and synergistically. Cellulases catalyze the hydrolysis of β-1,4-glycosidic bonds in cellulose.^56^ Exoglucanases and endoglucanases target the ends and randomly selected internal regions of the amorphous areas of cellulose, generating cello-oligosaccharides of varying lengths. Glucosidases subsequently hydrolyze these oligosaccharides into glucose.^57^ The glucose is then metabolized into CO_2_ through a series of downstream biochemical pathways. Previous studies have demonstrated that fungi, certain bacterial species, and actinomycetes found in compost and soil environments can produce cellulase and play a central role in cellulose degradation.^58^

#### 3.2.2 PLA-SL vs PLA-3L

The biodegradation of PLA (SL and 3L) showed three phases of biodegradation (Figure 3b): lag phase, biodegradation, and plateau. A lag phase of around 20 days is attributed to the initial abiotic hydrolysis phase. Ester bonds of PLA are cleaved during the hydrolytic abiotic degradation phase due to its susceptibility to water. As hydrolytic degradation proceeds, PLA chains are broken into smaller fragments, releasing small *M*_n_ oligomer populations. These lactic acid oligomers are available for microbial assimilation, releasing CO_2_ and water, which can be observed during the biotic degradation phase. A maximum CO_2_ evolution of 41.5 g and a biodegradation of 95% were observed for PLA-SL over the test duration (Figure 3b). No significant differences between PLA and PLA/PLA/PLA were observed in the lag or hydrolysis phases. According to Castro-Aguirre et al.,^59^ a *M*_n_ below 10 kDa is required before microbial assimilation can occur, following the completion of hydrolysis. Therefore, biodegradation starts when the *M*_n_ value of PLA declines below 10 kDa. The lower values of CO_2_ evolution before day 20 in the case of PLA indicate that the samples are still undergoing hydrolysis and are yet to be reduced to a point (≲10 kDa) needed to activate the biodegradation stage, where the microorganisms can start assimilating low *M*_n_ PLA, such as oligomers, dimers, and monomers, for their biochemical processes.^5^ Due to the higher thickness of the multilayer films (PLA-3L), biodegradation progresses more slowly after 20 days and reaches 90% at day 90.

#### 3.2.3 Effect of TPCS

PLA-*g*-TPCS, whatever the nature of the plasticizer, did not show any lag phase compared to the 20-day phase for PLA (Figure 3d). This finding can be attributed to the presence of TPCS, which serves as an initial nutrient source for microorganisms before PLA undergoes hydrolysis, fragmentation, and depolymerization into smaller, more absorbable form units. Like cellulose, starch is a natural, hydrophilic, and biodegradable polymer readily consumed as microorganisms adapt to the composting environment. The hydroxyl groups in TPCS facilitate and accelerate the biodegradation of PLA by promoting the disintegration of PLA-*g*-TPCS films. Numerous studies have demonstrated that incorporating starch into a polymer matrix enhances its water absorption capacity, further contributing to its degradation, they displayed that PLA-*g*-TPCS fully biodegrade much faster than PLA.^5^

Maran et al. reported enhanced water absorption in starch-containing samples subjected to soil burial degradation tests.^60^ Including starch increases the hygroscopic nature of films, promoting water uptake and creating favorable conditions for rapid hydrolysis, microbial invasion, and colonization.^61^ Additionally, the branched hydroxylated amylopectin chains facilitate deeper water penetration within the PLA-*g*-TPCS film matrix.^62^

At high initial *M*_w_, there is restricted segmental mobility of the backbone chains of PLA, and as such, less access to the hydroxyl and hydrophilic terminal carboxyl groups by water. However, the presence of the plasticizer in the films may act as a catalyst for water diffusion and sorption into the PLA matrix. The low initial *M*_w_ of plasticized TPCS films is assumed to be an advantage for a fast-starting *M*_w_ reduction compared to neat PLA films. This sensitivity of starch to water assists in the enzymatic hydrolysis to glucose. This is previously supported by observed changes in mechanical and water barrier properties as well as surface roughness and contact angles of PLA and PLA-*g*-TPCS films following the addition of TPCS, as illustrated in Table S7 in the Supporting Information. The addition of TPCS to PLA not only accelerates the disintegration of PLA-*g*-TPCS films under industrial composting conditions but also presents a promising opportunity to render PLA blends biodegradable in home composting settings, provided that key parameters, such as aeration and moisture, are appropriately managed.

PLA-*g*-TPCS films plasticized with glycerol showed a CO_2_ evolution of 42 g and a maximum mineralization of 95% over the test duration (Figure 3c and d). On the other hand, in the case of reactive blend plasticized with PEG, it reached a CO_2_ evolution of 36 g and a maximum mineralization of 96% at 90 days (Figure 3c and d). The presence of PEG showed a lower initial biodegradation trend than glycerol (30% biodegradation on day 20 against 40% biodegradation for glycerol). This phenomenon can be attributed to the high tendency of the Gly plasticizer to migrate, a relatively small change in crystallinity degree, and chain scission of macromolecules, resulting in a straightforward diffusion process for water and oxygen that facilitates higher microbial and enzymatic attack. This may be due to the large *M*_n_ of PEG ∼1500 Da compared to glycerol.

#### 3.2.4 Single vs Multilayer Films

Figure 3e and f shows the CO_2_ evolution and mineralization of reactive blend PLA-*g*-TPCS plasticized with glycerol in a single-layer and multilayer structure. Single-layer PLA-*g*-TPCS-Gly evolves faster than that of the PLA/PLA-*g*-TPCS-Gly/PLA multilayer film. Biodegradation reaches 95% and 89%, respectively, for the single-layer and multilayer structures on day 90 (Figure 3f). The effect is more pronounced on day 20 (17% and 38%, respectively, for the multilayer and the single layer structure), and the rate of biodegradation (slope of biodegradation during the abiotic phase) is lower in the case of the multilayer film (before day 20). Both PLA outer layers protect the single layer PLA-*g*-TPCS, so water diffuses slowly and reduces the immediate evolution of CO_2,_ and, therefore, the biodegradation is slower. The hydroxyl groups in TPCS, which accelerate the biodegradation of PLA in the reactive blend, are masked by the PLA outer layer. The single-layer structure has a more hygroscopic nature, promoting water uptake and creating favorable conditions for rapid hydrolysis, microbial invasion, and colonization. Once microorganisms use starch as their food source, fragmented PLA films (holes and cracks) are left behind. These structural discrepancies, in the form of macroscopic fractures, facilitate the biodegradation process.

A similar trend was observed for the reactive blend plasticized with PEG (Figure 3g and h). Biodegradation reaches 90% and 95%, respectively, for the multilayer and the single-layer structures on day 90 (Figure 3h). Similarly, this finding can be attributed to TPCS, which acts as an initial nutrient source for microorganisms before the depolymerization of PLA into smaller and more assimilable units.^5^ PEG-plasticized TPCS is more hydrophilic (similar to cellulose) and accelerates polymer biodegradation in a single-layer structure compared to multilayer films protected by PLA outer layers.

Overall, the multilayer structure incorporating PLA-*g*-TPCS initially degraded faster than the PLA/PLA/PLA multilayer films, demonstrating that a middle layer of PLA-*g*-TPCS can slightly enhance the oxygen barrier properties of (Figure 3f and h). Additionally, this structure reduces the lag phase of PLA films during industrial composting, potentially paving the way for the development of home-compostable PLA structures.

Mineralization kinetics were modeled by using the Hill equation (eq 5), which captures the sigmoidal behavior characteristic of cooperative degradation processes. This approach provided a quantitative framework for distinguishing the degradation pathways of the different films with the fitted parameters reflecting onset, rate, and extent of mineralization, which are summarized in Table S9. *Deg*_max_, which represents the percentage of mineralization at infinite time,^63^ matched the plateau phase observed in Figure 3. Cellulose exhibited the highest maximum of biodegradation compared with PLA-*g*-TPCS plasticized with glycerol or PEG. It took less than 40 days for all the samples to reach 50% of *Deg*_max_, as indicated by *k* values (time when *Deg* = 1/2 *Deg*_max_). Cellulose had the lowest *k* value (3.72 ± 0.18), followed by PLA-*g*-TPCS plasticized with PEG (28.59 ± 0.84). A similar finding was reported by David et al. for cellulose.^63^ The multilayer structure exhibited mineralization values comparable to those of the PLA-SL film. The Hill constant *n*, which reflects the curvature of the sigmoidal function, was higher for PLA-SL and -3L structures than for the other materials. This indicates that the biodegradation onset occurred slightly later in these systems compared to cellulose and PLA-*g*-TPCS plasticized with Gly or PEG, likely due to greater accessibility of microbial enzymes in the latter. This behavior can be attributed to the TPCS component, which provides an initial nutrient source for microorganisms before the depolymerization of PLA into smaller, more assimilable fragments. Incorporating two outer PLA layers around the PLA-*g*-TPCS core further increased the *n* value, suggesting an enhanced resistance of the multilayer material to biodegradation. Moreover, the time required to reach the maximum biodegradation rate (Time_rate max_; data not shown) was shorter for cellulose and PLA-*g*-TPCS-Gly (or PEG) than for PLA-SL and -3L films. It should be noted that the Hill model does not fully capture the kinetics of reactive blends composed of hydrophobic and hydrophilic matrices, underscoring the need for further studies to elucidate the underlying degradation pathways.

## 4 Conclusions

The production and characterization of reactive blends of PLA-*g*-TPCS in single-layer and multilayer structures were evaluated. MA as a compatibilizer and DCP as an initiator enhanced interfacial adhesion between PLA and TPCS, confirmed by FTIR spectra showing successful grafting and polymerization. Reactive PLA-*g*-TPCS films were more extensible but had lower strength and stiffness than neat PLA due to the ductility of TPCS. Multilayer structures exhibited greater resistance than monolayer films, with the TPCS inner layer improving oxygen barrier properties. However, incorporating plasticized TPCS slightly increased the WVP. Migration of plasticizers to the surface also decreased the contact angle, reflecting increased hydrophilicity. The hydrophilic nature of TPCS and its role as a nutrient source accelerated the degradation of PLA (i.e., PLA hydrolysis during the lag phase under thermophilic conditions due to increased chain mobility). The acceleration of the biodegradation rate depends on the nature of the plasticizers. Using the PLA-*g*-TPCS middle layer enhances the biodegradation of the PLA multilayer, paving the way for the production of home-compostable structures based on PLA. However, further research is needed to develop commercial films that are tougher and more compostable. Future work should explore the production of films using a one-step continuous process, as the initial stage, involving twin-screw extrusion in reactive blending, contributes significantly to reducing the *M*_w_ of PLA. Additionally, subsequent research should focus on elucidating how the incorporation of plasticized TPCS influences the barrier properties and compostability of PLA-based films.

## Supplementary Material



## Notes

Table S1 provides soil compost testing results for the compost used in the biodegradation experiments. Table S2 shows the carbon content analysis of the produced samples. Table S3 reports the thickness (μm) of each film layer obtained by digital microscope analysis. Table S4 lists the tensile properties (TS, YM, and EAB) of the single- and three-layer films. Table S5 contains the number-average molecular weight (*M*_n_), weight-average molecular weight (*M*_w_), and polydispersity index (PI) of the PLA layers determined by GPC. Table S6 summarizes the thermal parameters: glass transition temperature (*T*_g_), crystallization temperature (*T*_c_), melting temperature (*T*_m_), and degree of crystallinity (*X*_c_) from the second DSC heating cycle as well as the onset temperature (*T*_onset_), maximum degradation temperature (*T*_dmax_), and residue from TGA analysis. Table S7 reports the water contact angle (θ, °) for single- and three-layer films. Table S8 provides the biodegradation percentages at 45, 60, and 90 days, and Table S9 lists the Hill model parameters (*Deg*_max_, *k*, *n*) for the biodegradation kinetics of single- and multilayer films. Figure S1 shows the optical transmittance (%) as a function of wavelength (nm), Figure S2 displays the full FTIR spectra (500–4000 cm^–1^) in absorbance mode as a function of wavenumber (cm^–1^), Figure S3 presents the DMA thermograms, Figure S4 displays the DSC for the first heating cycle of the films, Figure S5 presents the TGA thermograms of the films, and Figure S6 illustrates the molecular weight distribution (MWD) curves [d*w*/d­(log *M*_w_) vs log­(*M*_w_)] obtained from GPC data (PDF)
