# Supplementary material for: Compostable Multilayer Films with Tailored Gas Barrier and Biodegradation
Source: ACS Omega. 2026 Mar 6;11(10):15869–83. doi: 10.1021/acsomega.5c09487 (PMC13000776; doi:10.1021/acsomega.5c09487)
Supplement: Supplementary file 1 [file ao5c09487_si_001.pdf]

Supporting information for:

Compostable Multilayer Films with Tailored Gas Barrier and Biodegradation

Nasreddine BENBETTAIEB[1,2,], Anibal BHER[3], Pooja C. MAYEKAR[3], Wanwarang LIMSUKON[3,4], Rafael AURAS[3,*]*

1. University Burgundy Europe, Institut AgroDijon, INRAE, UMR PAM, Food Processing and Microbiology, 1 Esplanade Erasme, 21000 Dijon, France.
2. University Burgundy Europe, IUT-Dijon, BioEngineering dpt., 7 Blvd Docteur Petitjean, BP17867, 20178 Dijon Cedex, France.
3. School of Packaging, Michigan State University, East Lansing, Michigan 48824, USA
4. Department of Food Science and Technology, Faculty of Science and Technology, Rajamangala University of Technology Tawan-ok, Sriracha, Chonburi 20110, Thailand

* Corresponding authors: Nasreddine BENBETTAIEB (E-mail: nasreddine.benbettaieb@u-bourgogne.fr) & Rafael AURAS (E-mail: aurasraf@msu.edu)

Table of Contents for Figures and Tables

Table S1. Soil compost testing for the compost used for the biodegradation test. 3
Table S2. Carbon analysis for the evaluated samples. 4
Table S3. Thickness (µm) of each layer of the films using digital microscope analysis. 5
Table S4. Tensile properties (TS, YM and EAB) of single and three-layer films evaluated. 7
Table S5. M_n, M_w, and PI of the PLA layer of the single and three-layer films produced by cast film extrusion. 9
Table S6. Values of T_g, T_c, T_m, and X_c from the second heating cycle of differential scanning calorimetry (DSC), and T_{onset}, T_{dmax} and residue determined from TGA analysis for single and three-layer films..........10
Table S7. Water contact angle (°) for single and three-layer films...12
Table S8. Biodegradation % of samples at day 45, 60, and 90. 13
Table S9. Hill parameters (Deg $_{max}$, k, n) of single and multilayer films. 14

Figure S1. Transmittance (%) as a function of wavelength (nm) for a) single PLA, PLA-*g*-TPCS-GLY, and PLA-*g*-TPCS_PEG layer and b) the three-layer PLA/PLA/PLA, PLA/PLA-*g*-TPCS-Gly/PLA, and PLA/PLA-*g*-TPCS-PEG/PLA films. 5
Figure S2. FTIR spectra in absorbance mode as a function of wave number (Full spectra: 500-4000 cm^{-1}) of PLA, PLA/PLA/PLA, PLA-*g*-TPCS-Gly, PLA/PLA-*g*-TPCS-Gly/PLA, PLA-*g*-TPCS-PEG, and PLA/PLA-*g*-TPCS-PEG/PLA films. 6
Figure S3. Dynamic mechanical analysis (DMA) thermograms of single and three-layer films: (a) *G*', storage modulus; (b) *G*", loss modulus; and (c) tan (*δ*). 8
Figure S4. DSC thermograms of the first heating cycle of single and three-layer films produced by cast film extrusion 11
Figure S5. TGA thermogram of single and three-layer films produced by cast film extrusion, a) single PLA, PLA-*g*-TPCS-Gly, and PLA-*g*-TPCS_PEG layer and b) the three-layer PLA, PLA/PLA-*g*-TPCS-Gly/PLA, and PLA/PLA-*g*-TPCS-PEG/PLA films. 12
Figure S6. Molecular weight distribution (MWD): dw/d(log *M*w) vs log (*M*w) for a) a) PLA, PLA-g-TPCS-Gly, and PLA/PLA-*g*-TPCS-Gly/PLA, b) PLA, PLA-g-TPCS-PEG, and PLA/PLA-*g*-TPCS-PEG/PLA films. 15

Section S1

Below results for physicochemical properties of the compost used for biodegradation testing.

Table S1. Soil compost testing for the compost used for the biodegradation test.

Tests	Units	Results
pH		7.28
E. C. - Saturation Paste	mmho/cm	11.2
Total Dry Solid	%	57.5
Total Volatile Solid	%	48.8
C/N Ratio		10.5
Total Nitrogen (N)	%	2.80
Total Phosphorus (P)	%	1.03
Total Potassium (K)	%	2.11
Total Calcium (Ca)	%	4.93
Total Magnesium (Mg)	%	1.81
Total Zinc (Zn)	ppm	298
Total Iron (Fe)	ppm	2844
Total Manganese (Mn)	ppm	346
Total Copper (Cu)	ppm	132
Total Carbon (C)	%	29.4
Total Sodium (Na)	%	0.394
Total Aluminum (Al)	%	0.263
Total Sulfur (S)	%	0.531
Total Boron (B)	ppm	55.9

*Interpretation for nitrate-N is for growing media only. If this material is to be used as soil amendment, the interpretation for nitrate-N is not applicable.

Below the carbon elemental analysis for films samples evaluated during the biodegradation test.

Table S2. Carbon analysis for the evaluated samples.

Material	Carbon (%)*
Cellulose	42.42 ± 1.17
PLA	50.40 ± 0.9
PLA/PLA/PLA	50.40 ± 0.9
PLA-*g*-TPCS-Gly	51.96 ± 4
PLA/ PLA-*g*-TPCS-Gly/PLA	50.89 ± 3.1
PLA-*g*-TPCS-PEG	52.96 ± 0.1
PLA/PLA-*g*-TPCS-PEG/PLA	51.34 ± 0.9

* Percentage by weight.

Section S2

Below results for characterization of single and multilayer films.

Table S3. Thickness (µm) of each layer of the films using digital microscope analysis.

Formulation	Thickness (µm)
PLA	12.31 ± 0.58[a]
PLA/PLA/PLA	41.96 ± 1.38[b]
PLA/PLA/PLA (inner layer)	10.99 ± 2.53[a]
PLA-*g*-TPCS-Gly	26.66 ± 0.65[c]
PLA/PLA-*g*-TPCS-Gly/PLA	41.17 ± 1.16[b]
PLA/PLA-*g*-TPCS-Gly/PLA (inner layer)	11.12 ± 2.44[a]
PLA-*g*-TPCS-PEG	16.72 ± 2.18[c]
PLA/PLA-*g*-TPCS-PEG/PLA	35.8 ± 1.34[d]
PLA/PLA-*g*-TPCS-PEG/PLA (inner layer)	9.35 ± 1.48[a]

Note: Mean ± std deviation. Values in a column with the same superscript letter are not significantly different at p-level = 0.05.

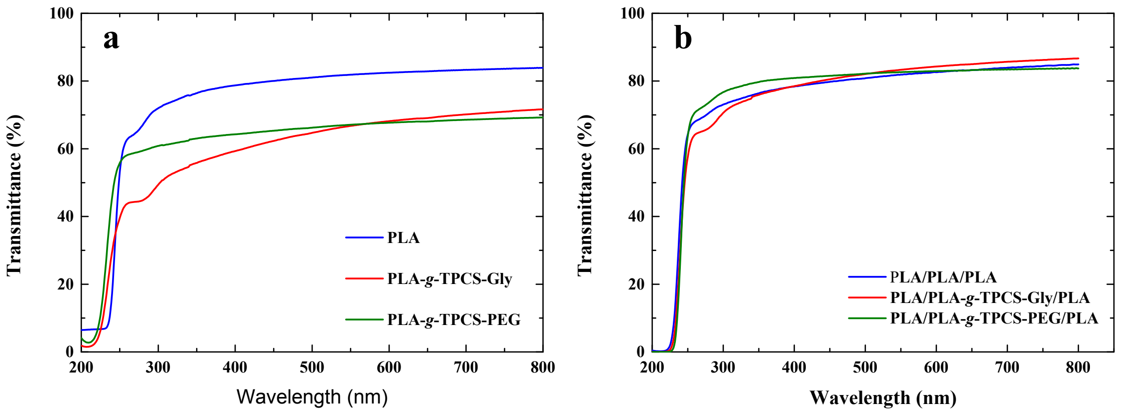

Figure S1. Transmittance (%) as a function of wavelength (nm) for a) single PLA, PLA-*g*-TPCS-GLY, and PLA-*g*-TPCS_PEG layer and b) the three-layer PLA/PLA/PLA, PLA/PLA-*g*-TPCS-Gly/PLA, and PLA/PLA-*g*-TPCS-PEG/PLA films.

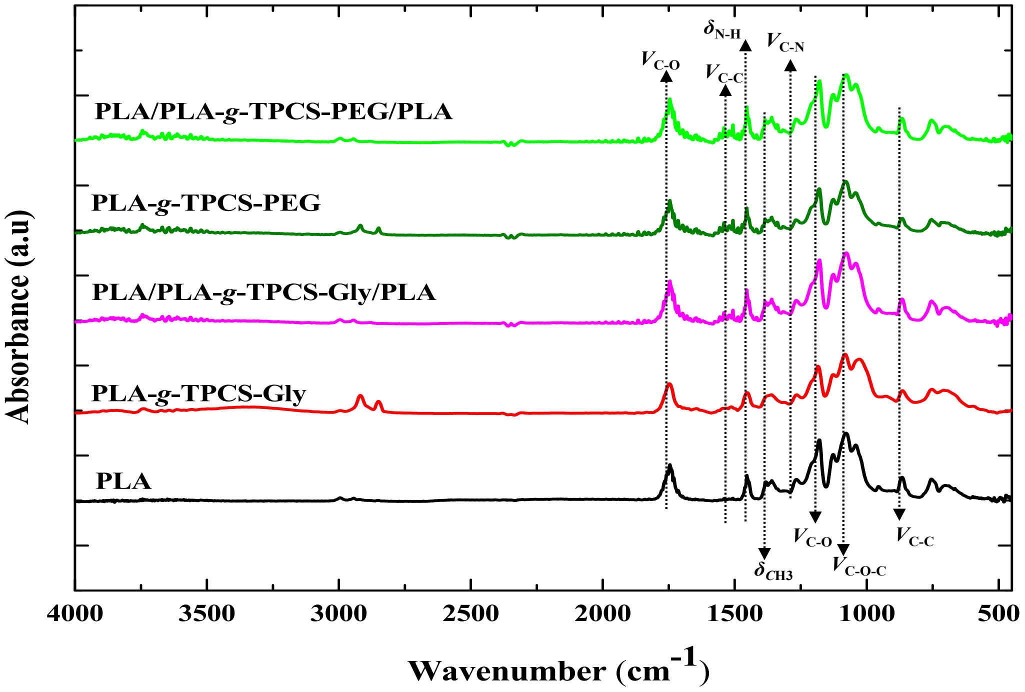

Figure S2. FTIR spectra in absorbance mode as a function of wave number (Full spectra: 500-4000 cm^{-1}) of PLA, PLA/PLA/PLA, PLA-*g*-TPCS-Gly, PLA/PLA-*g*-TPCS-Gly/PLA, PLA-*g*-TPCS-PEG, and PLA/PLA-*g*-TPCS-PEG/PLA films.

Table S4. Tensile properties (TS, YM and EAB) of single and three-layer films evaluated.

Formulation	Thickness (μm)	TS (MPa)	YM (MPa)	EAB (%)
PLA	12.31 ± 0.58^{a}	44.83 ± 1.91^{a}	2806.4 ± 156.13^{a}	6.75 ± 1.52^{a}
PLA/PLA/PLA	41.96 ± 1.38^{b}	45.2 ± 2.74^{a}	2754 ± 150.5^{a}	9.41 ± 1.45^{a}
PLA-*g*-TPCS-Gly	26.66 ± 0.65^{c}	14.32 ± 1.63^{b}	1033.7 ± 118.83^{b}	20.91 ± 3.01^{b}
PLA/PLA-*g*-TPCS-Gly/PLA	41.17 ± 1.16^{b}	40.98 ± 4.19^{a}	2346.5 ± 110.58^{c}	6.71 ± 1.37^{a}
PLA-*g*-TPCS-PEG	16.72 ± 2.18^{d}	9.52 ± 1.37^{c}	726.3 ± 80.38^{d}	26.56 ± 3.1^{c}
PLA/PLA-*g*-TPCS-PEG/PLA	35.8 ± 1.34^{e}	33.9 ± 2.59^{d}	2434.5 ± 112.54^{c}	8.22 ± 2.06^{a}

Note: Mean ± std deviation. Values in a column with the same superscript letter are not significantly different at *p*-level = 0.05 (Tukey's test).

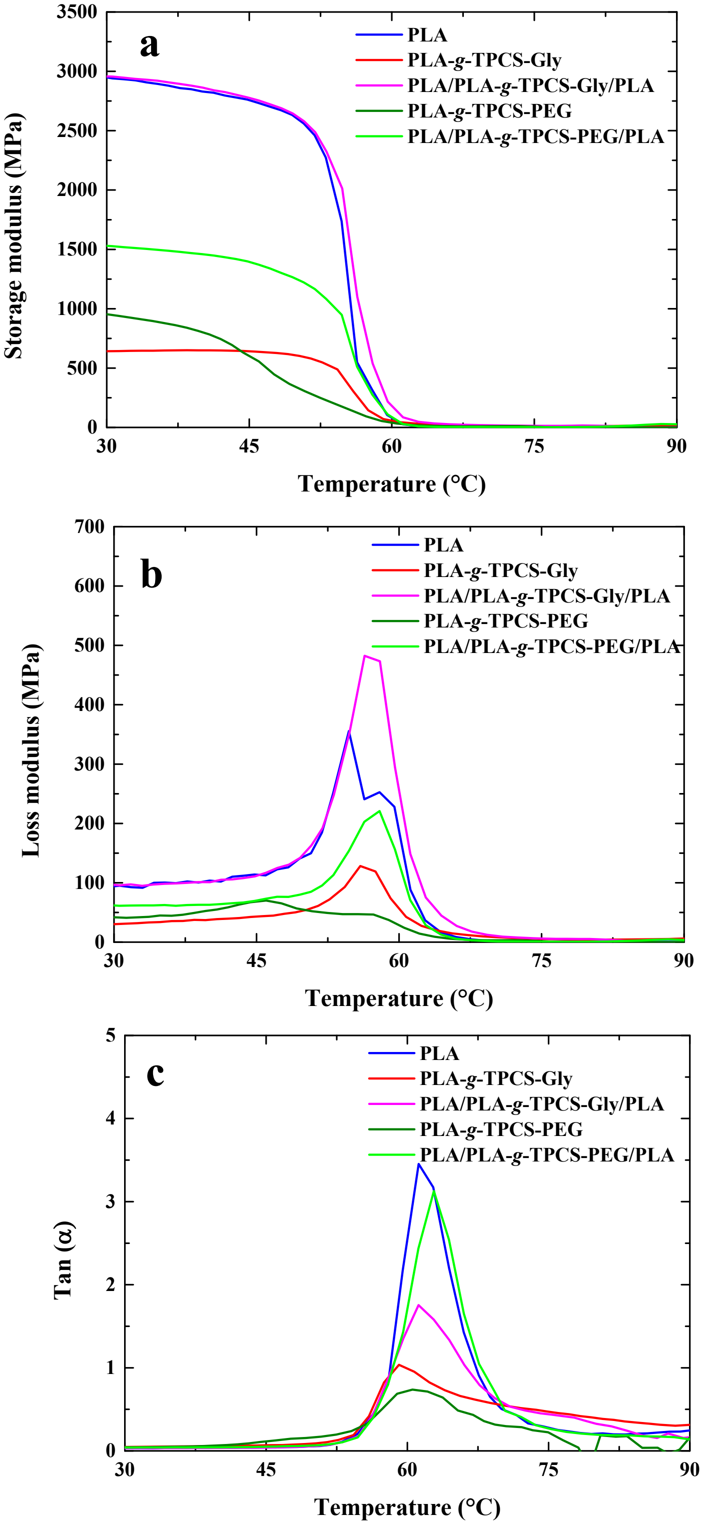

Figure S3. Dynamic mechanical analysis (DMA) thermograms of single and three-layer films: (a) *G*', storage modulus; (b) *G*", loss modulus; and (c) tan (δ).

Table S5. M_n, M_w, and PI of the PLA layer of the single and three-layer films produced by cast film extrusion.

Formulation	M_n (kDa)	M_w (kDa)	PI
PLA	56.9 ± 8.8[a]	101.1 ± 16.9[a]	1.77 ± 0.028[a]
PLA-*g*-TPCS-Gly	36.6 ± 1.7[b]	61.7 ± 0.5[b]	1.68 ± 0.085[b]
PLA/PLA-*g*-TPCS-Gly/PLA	52.1 ± 1.8[a]	87.8 ± 1.1[a]	1.68 ± 0.38[b]
PLA-*g*-TPCS-PEG	43.9 ± 3.0[c]	75.6 ± 2.6[c]	1.72 ± 0.084[c]
PLA/PLA-*g*-TPCS-PEG/PLA	41.5 ± 0.2[c]	67.2 ± 1.3[d]	1.61 ± 0.028[d]

Mean ± std deviation. Values in a column with the same superscript letter are not significantly different at p-level = 0.05.

Table S6. Values of T_g, T_c, T_m, and X_c from the second heating cycle of differential scanning calorimetry (DSC), and T_{onset}, T_{dmax} and residue determined from TGA analysis for single and three-layer films.

Formulation	T_g from DSC (°C)	T_g from DMA (°C)	T_c (°C)	T_m (°C)	X_c (%)	$T_{onset,}$ °C	T_{dmax} °C	Residue, %
PLA	57.2 ± 1.94^{a}	61.81 ± 0.58^{a}	125 ± 1.3^{a}	150.7 ± 0.9^{a}	0.31 ± 0.23^{a}	320 ± 2^{a}	367.64 ± 1.71^{a,b}	0.027 ± 0.038^{a}
PLA/PLA/PLA								
PLA-*g*-TPCS-Gly	54.98 ± 2.84^{a}	59.56 ± 0.41^{b}	112 ± 2^{b}	142.2 ± 0.5^{b} 147.1±0.3^{c}	7.88 ± 0.55^{b}	308 ± 1.2^{b}	365.5 ± 0.4^{a}	0^{a}
PLA/PLA-*g*-TPCS-Gly/PLA	55.17 ± 0.29^{a}	61.98 ± 1.11^{a,c}	95 ± 0.5^{c}	152.5 ± 1.2^{a}	21.44 ± 0.81^{c}	315.7 ± 0.5^{c}	369.59 ± 0.55^{b}	2.37 ± 3.35^{a}
PLA-*g*-TPCS-PEG	41.27 ± 1.68^{b}	58.79 ± 2.98^{a,b}	89 ± 1.7^{d}	140.1 ± 0.9^{b} 151.1± 0.4^{a}	2.19 ± 0.4^{d}	315.4 ± 1.3^{c}	366.62 ± 0.77^{a}	0.026 ± 0.036^{a}
PLA/PLA-*g*-TPCS-PEG/PLA	56.38 ± 2.96^{a}	62.81 ± 0^{c}	126 ± 1.1^{a}	153.6 ± 1.1^{a}	13.35 ± 5.78^{e}	319.33 ± 0.3^{a}	370.33 ± 0.6^{b}	1.99 ± 2.81^{a}

Mean ± std deviation. Values in a column with the same superscript letter are not significantly different at p-level = 0.05.

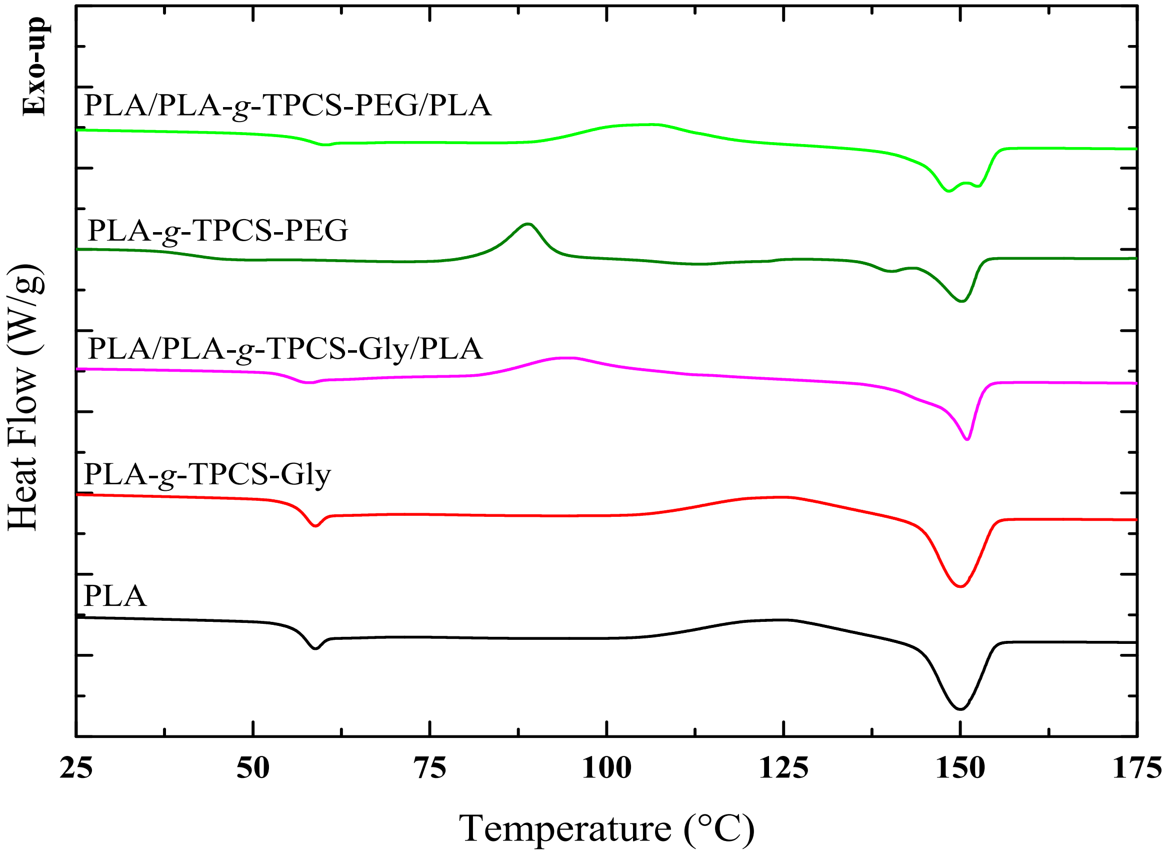

96

97 **Figure S4**. DSC thermograms of the first heating cycle of single and three-layer films produced by cast film extrusion

98

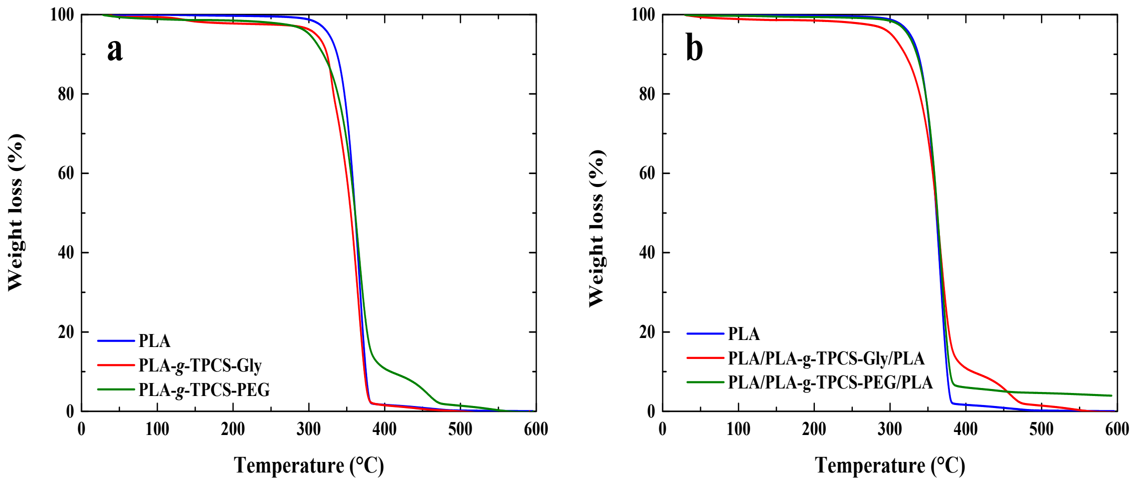

Figure S5. TGA thermogram of single and three-layer films produced by cast film extrusion, a) single PLA, PLA-*g*-TPCS-Gly, and PLA-*g*-TPCS_PEG layer and b) the three-layer PLA, PLA/PLA-*g*-TPCS-Gly/PLA, and PLA/PLA-*g*-TPCS-PEG/PLA films.

Table S7. Water contact angle (°) for single and three-layer films

Formulation	Water contact angle (°)
PLA	73 ± 1[a]
PLA-*g*-TPCS-Gly	64 ± 4[b]
PLA/PLA-*g*-TPCS-Gly/PLA -Face 1	66.7 ± 3.3[b]
PLA/PLA-*g*-TPCS-Gly/PLA -Face 2	76 ± 1.7[a]
PLA-*g*-TPCS-PEG	67 ± 3.8[b]
PLA/PLA-*g*-TPCS-PEG/PLA -Face 1	66.7 ± 3.3[b]
PLA/PLA-*g*-TPCS-PEG/PLA -Face 2	75.6 ± 1.5[a]

Mean ± std deviation. Values in a column with the same superscript letter are not significantly different at p-level = 0.05.

Table S8. Biodegradation % of samples at day 45, 60, and 90.

Formulation	Biodegradation at day 45 (%)	Biodegradation at day 60 (%)	Biodegradation at day 90 (%)
Cellulose	104.5 ± 6.9^{a}	103.5 ± 7.5^{a}	101.7 ± 9.5^{a}
PLA	78.0 ± 9.5^{b}	94.7 ± 11.4^{a,b}	95.4 ± 15.3^{a,b}
PLA/PLA/PLA	69.1 ± 10.7^{b}	85.2 ± 13.5^{b}	90.1 ± 18.0^{b}
PLA-*g*-TPCS-Gly	76.9 ± 7.0^{b}	95.8 ± 8.3^{a,b}	95.2 ± 11.1^{a,b}
PLA/PLA-*g*-TPCS-Gly/PLA	66.1 ± 6.5^{b}	84.3 ± 8.1^{a,b}	89.1 ± 10.3^{b}
PLA-*g*-TPCS-PEG	81.4 ±18.7^{a,b}	93.8 ± 22.2^{a,b}	95.9 ± 29.4^{a,b}
PLA/PLA-*g*-TPCS-PEG/PLA	70.0 ± 3.0^{b}	83.0 ± 3.2^{a,b}	89.9 ± 4.0^{b}

Mean ± std deviation. Values in a column with the same superscript letter are not significantly different at p-level = 0.05.

Table S9. Hill parameters (Deg $_{max}$, k, n) of single and multilayer films.

Sample	Parameters		
	Deg_{max}, %	k, d	n
Cellulose	104.6 ± 0.7^{a}	3.72 ± 0.18^{a}	2.15 ± 0.19^{b}
PLA	99.3 ± 1.9^{bc}	33.56 ± 0.67^{bc}	4.53 ± 0.38^{c}
PLA/PLA/PLA	95.4 ± 2.8^{c}	36.77 ± 1.00^{c}	4.34 ± 0.46^{c}
PLA-g-TPCS-Gly	137.3 ± 11.3^{c}	38.44 ± 5.47^{c}	1.32 ± 0.12^{a}
PLA-g-TPCS-PEG	109.4 ± 2.3^{b}	28.59 ± 0.84^{b}	2.15 ± 0.11^{b}
PLA/ PLA-g-TPCS-Gly/PLA	104.1 ± 3.5^{c}	36.30 ± 1.34^{c}	2.56 ± 0.18^{b}
PLA/ PLA-g-TPCS-PEG/PLA	103.2 ± 1.5^{bc}	34.74 ± 0.58^{bc}	2.50 ± 0.08^{b}

Values with different letters in a column are statistically different ($\alpha = 0.05$ Tukey–Kramer Test).

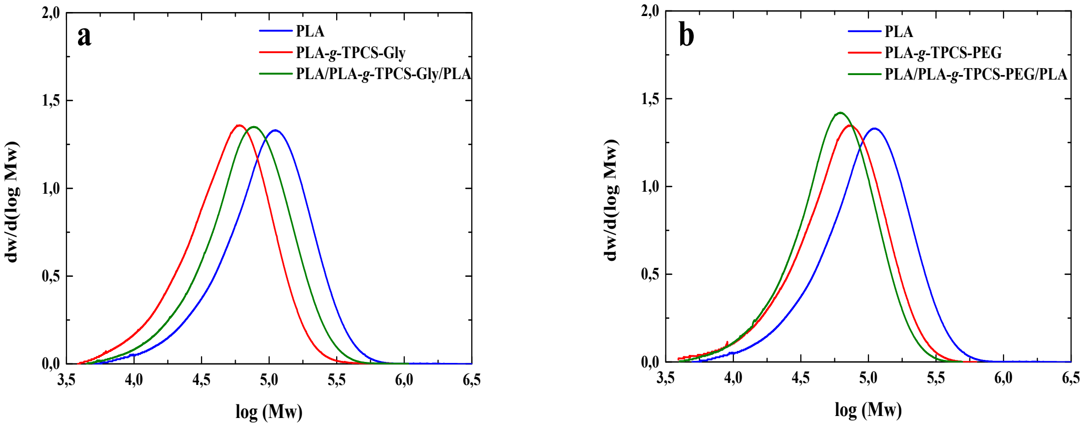

Figure S6. Molecular weight distribution (MWD): dw/d(log *M*w) vs log (*M*w) for a) a) PLA, PLA-g-TPCS-Gly, and PLA/PLA-*g*-TPCS-Gly/PLA, b) PLA, PLA-g-TPCS-PEG, and PLA/PLA-*g*-TPCS-PEG/PLA films.
